# Educational and social inequalities and cause-specific mortality: a prospective study in two municipalities of Mexico City

**DOI:** 10.1016/S2468-2667(23)00153-6

**Published:** 2023-09

**Authors:** Thomas Addey, Jesus Alegre-Díaz, Fiona Bragg, Eirini Trichia, Rachel Wade, Rogelio Santacruz-Benitez, Raúl Ramirez-Reyes, Adrian Garcilazo-Ávila, Carlos Gonzáles-Carballo, Omar Yaxmehen Bello-Chavolla, Neftali Eduardo Antonio-Villa, Diego Aguilar-Ramirez, Louisa Gnatiuc Friedrichs, Sarah Lewington, Richard Peto, Rory Collins, Jaime Berumen, Jonathan R Emberson, Pablo Kuri-Morales, Roberto Tapia-Conyer

**Affiliations:** 1 Clinical Trial Service Unit and Epidemiological Studies Unit, Nuffield Department of Population Health, University of Oxford, Oxford, UK; 2 Experimental Research Unit from the Faculty of Medicine, National Autonomous University of Mexico, Mexico City, Mexico; 3 MRC Population Health Research Unit, Nuffield Department of Population Health, University of Oxford, Oxford, UK; 4 Research Division, Instituto Nacional de Geriatría, Mexico City, Mexico; 5 Department of Endocrinology, Instituto Nacional de Cardiología Ignacio Chávez, Mexico City, Mexico; 6 School of Medicine, National Autonomous University of Mexico, Mexico City, Mexico; 7 Instituto Tecnológico y de Estudios Superiores de Monterrey, Monterrey, Mexico

## Abstract

**Background:**

Social inequalities in adult mortality have been reported across diverse populations, but there is no large-scale prospective evidence from Mexico. We aim to quantify social, including educational, inequalities in mortality among adults in Mexico City.

**Methods:**

The Mexico City Prospective Study recruited 150 000 adults aged 35+ years from Mexico City between 1998 and 2004. Participants were followed-up until 1 January 2021 for cause-specific mortality. Cox regression analysis yielded rate ratios (RRs) for death at ages 35-74 associated with education and examined, in exploratory analyses, the mediating effects of lifestyle and related risk factors.

**Findings:**

Among 143 478 participants aged 35-74, there was a strong inverse association of education with premature death. Compared with participants with tertiary education, after adjustment for age and sex, those with no education had about twice the mortality rate (RR 1.84 [95% CI 1.71-1.98]), equivalent to approximately 6 years lower life expectancy, with RR of 1.78 (1.67-1.90), 1.62 (1.53-1.72) and 1.34 (1.25-1.42) among participants with incomplete primary, complete primary and secondary education, respectively. Education was most strongly associated with death from renal disease and acute diabetic crises (RR 3.65 [95% CI 3.05-4.38] for none vs. tertiary education) and from infectious disease (2.67 [2.00-3.56]), but there was an apparent higher rate of death from all specific causes studied with lower education. Lifestyle factors (smoking, alcohol drinking, leisure time physical activity) and related physiological correlates (adiposity, diabetes, blood pressure) accounted for 84% of the association of education with premature mortality.

**Interpretation:**

In this Mexican population there were marked educational inequalities in premature adult mortality, which appeared to largely be accounted for by lifestyle and related risk factors. Effective interventions to reduce these risk factors could reduce inequalities and have a major impact on premature mortality.

**Funding:**

Wellcome Trust, the Mexican Health Ministry, the National Council of Science and Technology for Mexico, Cancer Research UK, British Heart Foundation, and the UK Medical Research Council Population Health Research Unit

## Introduction

Social inequalities in health have been observed in many populations, although their nature and extent differ.^1,2^ Studies in Europe and North America have repeatedly shown higher mortality among more socially disadvantaged groups.^1,3,4^ Qualitatively similar social gradients in mortality have been reported in Latin American populations,^5–9^ and one of the few large-scale prospective studies from this region reported an almost doubling of premature mortality rates among Cuban adults who had not completed primary education when compared with those with university education.^7^ However, detailed understanding of the size and nature of social inequalities in mortality in other Latin American populations is lacking, reflecting limitations of study designs (e.g., ecological studies,^8^ small study populations^6^) and data (e.g., reliance on routine data^5,6^) used to investigate them. Reported prospective associations of education, or other socioeconomic indicators, with mortality among the Mexican population are limited to small-scale studies, generating mixed findings.^10–12^ Large-scale evidence is restricted to that from studies reliant on limited routine data, precluding detailed understanding of the nature of inequalities or factors contributing to them.^13^ Given the complexity of social patterning of health and disease, and influences—including context-dependent influences—on this, the generalisability of findings between regions and countries is unclear. Robust large-scale prospective evidence is needed to understand and mitigate social inequalities in health in the Mexican population.

We report the associations of education, as well as other socioeconomic indicators, with cause-specific mortality in a prospective cohort of 150 000 adults from Mexico City who have been followed for nearly two decades.

## Methods

### Study design and participants

The Mexico City Prospective Study (MCPS) design, methods and population have been described previously.^14^ Briefly, between 1998 and 2004, households in two districts of Mexico City (Coyoacán and Iztapalapa) were visited and household members aged ≥35 were invited to participate in the study. Of 112 333 households with eligible inhabitants, one or more individuals from 106 059 (94%) households consented to participate.^14^ Ethics approval was obtained from the Mexican Ministry of Health, the Mexican National Council for Science and Technology, and the University of Oxford, UK. All participants provided written informed consent.

### Data collection

During household visits, trained nurses administered electronic questionnaires collecting information on sociodemographic and lifestyle (including smoking, alcohol consumption and physical activity) factors, and measured height, weight, hip circumference (HC), waist circumference (WC) and sitting blood pressure using calibrated instruments and standard protocols. A non-fasting venous blood sample was collected into an EDTA vacutainer and separated into two plasma and one buffy coat aliquots for long-term storage at -150°C. HbA1c levels were measured in buffy coat samples using a validated high-performance liquid chromatography method^15^ on HA-8180 analysers with calibrators traceable to International Federation of Clinical Chemistry standards.^16^ A repeat survey performed between 2015 and 2019 and including approximately 10 000 surviving participants collected the same information as at the baseline survey.

### Assessment of socioeconomic position

Self-reported indicators of socioeconomic position included highest education level and personal (i.e., individual) monthly income. An area-based measure of social development—the Social Development Index (*Indice de Desarollo Social, 2010*)^17^—was obtained for each participant based on block of residence (determined from Global Positioning System coordinates recorded at the baseline household visit). The Social Development Index is a composite score calculated from block or *manzana* (each including just one or a small group of houses or buildings) level measures of six domains: quality and space of housing (33.8% weighting), access to health and social security (29.1%), education (24.4%), ownership of household goods (6.0%), and adequate access to water supply/drainage (3.8%) and energy (2.9%).^17^

### Follow-up for mortality

Participants are followed-up for cause-specific mortality through probabilistic linkage (based on name, including phonetic coding of names, age and sex [information on place of birth was not available]) to the Mexican System for Epidemiologic Death Statistics (Subsistema Epidemiológico y Estadístico de Defunciones or SEED) electronic death registry in Mexico City, administered by the Ministry of Health. Field validation of over 7000 matched deaths confirmed the reliability of the matching algorithm in >95% of deaths. Death registration in Mexico City is reliable and complete, with almost all deaths certified medically. Diseases recorded on death certificates are coded using the International Statistical Classification of Diseases and Related Health Problems, Tenth Revision, with subsequent review by study clinicians (unaware of baseline information) to recode, where necessary, the underlying cause of death.^15^ Participant deaths were tracked until 1 January 2021.

### Statistical analysis

Analyses excluded participants aged 85 or older, with missing education data, missing or extreme covariate data, or who had an uncertain cause of death (Table 1). Education was categorised to reflect Mexico’s main phases of schooling: none, incomplete primary, complete primary, secondary and tertiary education. Monthly income was grouped into five categories: no income reported, <1500 pesos, 1500 to <3000 pesos, 3000 to <4500 pesos, ≥4500 pesos (no income and 4 approximately equal groups). At the time of recruitment, 1500 pesos was equal to approximately 190 US dollars. The Social Development Index was subdivided into five groups by the quintiles of its distribution; higher scores represent higher social development, indicative of lower relative deprivation of an area.

Cox proportional hazards regression models, with time since entry into the study as the underlying timescale, were used to assess the relevance of education, income (among men only since 64% of women reported ‘no income’) and Social Development Index for all-cause and cause-specific mortality (Table S1). The log hazard ratio from a Cox model provides a useful summary statistic for the average log mortality rate ratio (RR) across the different time periods of follow-up. These mortality RRs were stratified by age-at-risk (5-year groups) and, for analyses of men and women combined, by sex. This allows for a different baseline hazard in each stratum, so the proportional hazards assumption was made only within strata of age-at-risk (and, where relevant, sex). Group-specific variances were estimated (reflecting the amount of data in each exposure category), such that the RR for each category, including the reference category, is associated with a group-specific 95% CI, enabling comparisons between any two categories and not only with the reference group.^18^ Participants who did not die from the cause of interest were censored at the earliest of death from any other cause, the end of the age-at-risk period of interest, or 31 December 2020. The main analyses examined premature mortality (i.e., deaths before 75 years^15^), but the relevance of education for mortality at 75-84 years was also examined.

Prospective cohort studies of non-representative cohorts of individuals—as in the current case of Mexican adults from just two Mexico City municipalities—can generate reliable evidence about the associations of risk factors with health outcomes that are generalisable to a wider underlying population.^19–21^ With this assumption, and also assuming causality and generalisability of the distributions of education and the causes of death in our study to the rest of Mexico, we estimated what the hypothetical effect might be of differing levels of education on survival from age 35 to 70 years at 2020 Mexican mortality rates. For example, if the national death rate for Mexican men in 2020^22^ for a given five-year age range was A, we calculated the death rates for each of the 5 education groups (i.e., tertiary, secondary, complete primary, incomplete primary and none) that would ensure that their prevalence-weighted average equalled A and the relative differences between them matched the RRs in our study.

The extent to which the association of education with mortality was accounted for by lifestyle (smoking status, alcohol consumption and leisure time physical activity) and related factors (adiposity [weight, height, WC and HC], diabetes status, and systolic blood pressure [SBP]) was assessed in exploratory analyses calculating the proportional change in the log-likelihood χ^2^ statistic and log RR when education was added to a model with and without potential mediators. The change in likelihood ratio statistic following inclusion of these factors (which may or may not be on the causal pathway) into the adjusted model provides a quantitative indication of the proportion of the association of education with mortality that can be attributed to these potential mediators.^23^ In these models, smoking status was categorised into five groups (never, former, occasional, <10 cigarettes/day, ≥10 cigarettes/day), alcohol consumption into five groups (never, former, less than weekly, up to two days per week, more than two days per week), physical activity into three groups (none, up to twice weekly, at least three times weekly), diabetes status into five groups (no diabetes; undiagnosed diabetes [no previous diabetes diagnosis and HbA1c >6.5%]; previously diagnosed diabetes with HbA1c <9%; previously diagnosed diabetes with HbA1c ≥9% and <11%; previously diagnosed diabetes with HbA1c ≥11%), and weight, height, WC, HC and SBP were included as continuous variables.

Analyses were conducted using SAS (version 9.4) and R (version 3.6.2).

### Role of the funding source

The funders had no role in study design, data collection, analysis or interpretation, or writing of the report. The corresponding authors had full access to all the data in the study and had final responsibility for the decision to submit for publication.

## Results

Of 159 517 participants recruited to the study, 2460 (2%) aged ≥85 at recruitment, a further 2808 (2%) with missing education data or missing or extreme covariate data, and another 1950 (1%) with uncertain mortality linkage were excluded. Overall, 152 299 participants remained for inclusion in the present analyses, including 143 478 (94%) aged 35-74 and 8821 (6%) aged 75-84 at recruitment.

**Figure 1:**
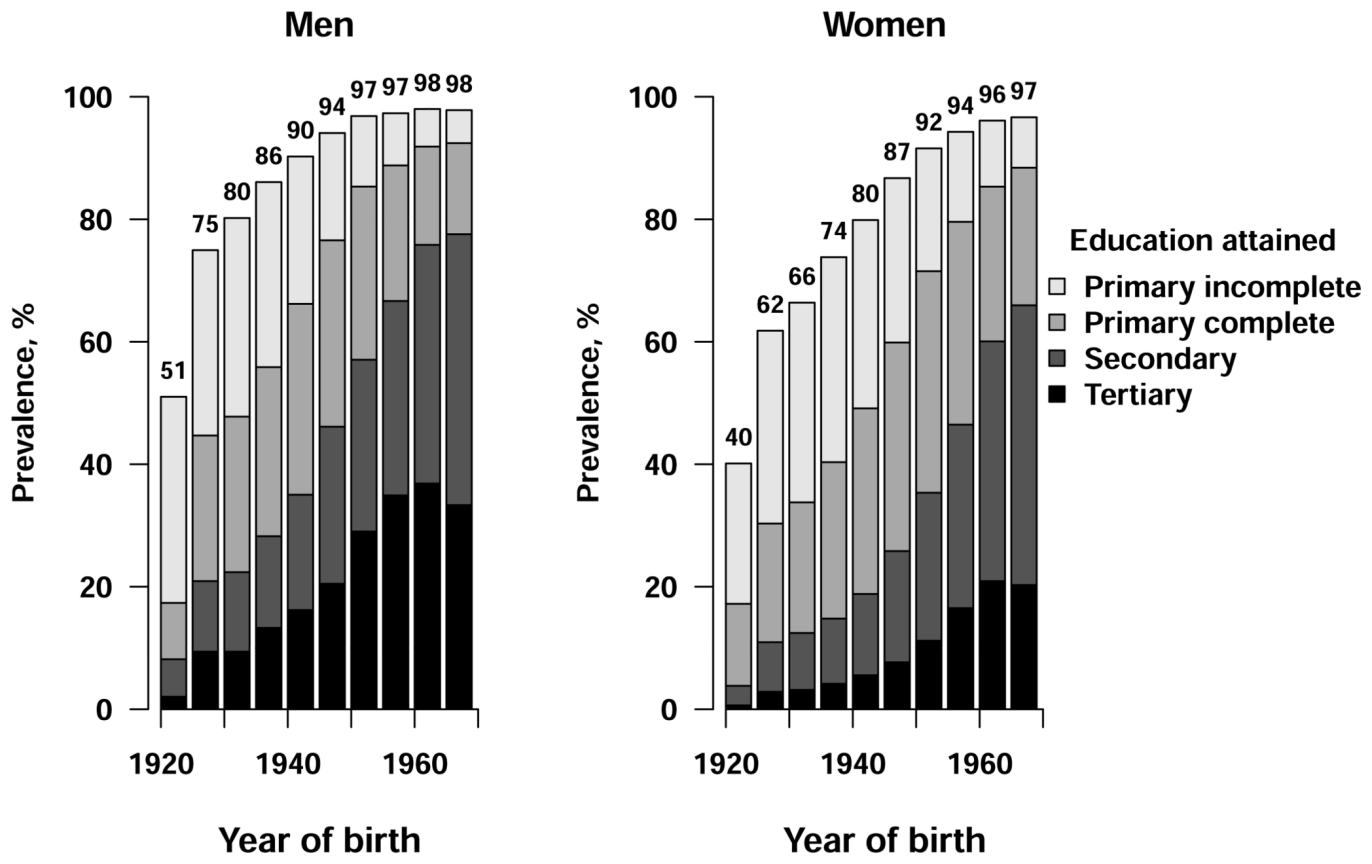
Education by year of birth Analyses among 143 478 participants aged 35-74 years at recruitment. Numbers above the bars are the percentage of participants in that birth cohort with any formal education.

Among participants aged 35-74 at recruitment, 33% (n=46 674) were men and the mean (SD) age was 50.7 (10.8) years (Table 1, Table S2). Men were, on average, more highly educated than women. Among men, 7% (n=3390) reported no education, 16% (n=7529) incomplete primary education, 24% (n=11 403) complete primary education, 27% (n=12 633) secondary education, and 25% (n=11 719) tertiary education. Corresponding proportions among women were 13% (n=12 358), 21% (n=20 083), 29% (n=28 257), 25% (n=24 313) and 12% (n=11 793), respectively. At recruitment, more highly educated participants tended to be younger, consistent with patterns of education by birth cohort (Figure 1). Higher levels of education were associated with higher monthly income, more frequent leisure time physical activity and higher prevalence of current alcohol drinking (Table 1). More highly educated women were more likely to be current or former smokers and to have lower adiposity levels. There was no clear trend in smoking status or adiposity according to education among men. Men and women with less education had higher blood pressure, more frequently reported a previous diagnosis of diabetes and, among those without a prior diagnosis of diabetes, tended to have higher HbA1c levels. The prevalence of other previously-diagnosed chronic diseases differed little by education. When 9941 participants were resurveyed, on average, 16 years after the baseline survey, self-reported education was unchanged among 72% (n=7185) (kappa=0.64 for agreement between baseline and resurvey) (Table S3).

During an average 18.4 (IQR 17.6-19.7) years of follow-up, 13,502 participants died at ages 35-74, including 3679 deaths from vascular disease (including 2585 cardiac and 800 stroke deaths), 1140 from hepatobiliary disease, 2710 from renal disease and acute diabetic crises, 2246 from cancer, 1779 from respiratory disease and 857 from infectious disease (Table S1). Among 152 299 participants aged 35-84 at recruitment, 7908 died at ages 75-84.

**Figure 2:**
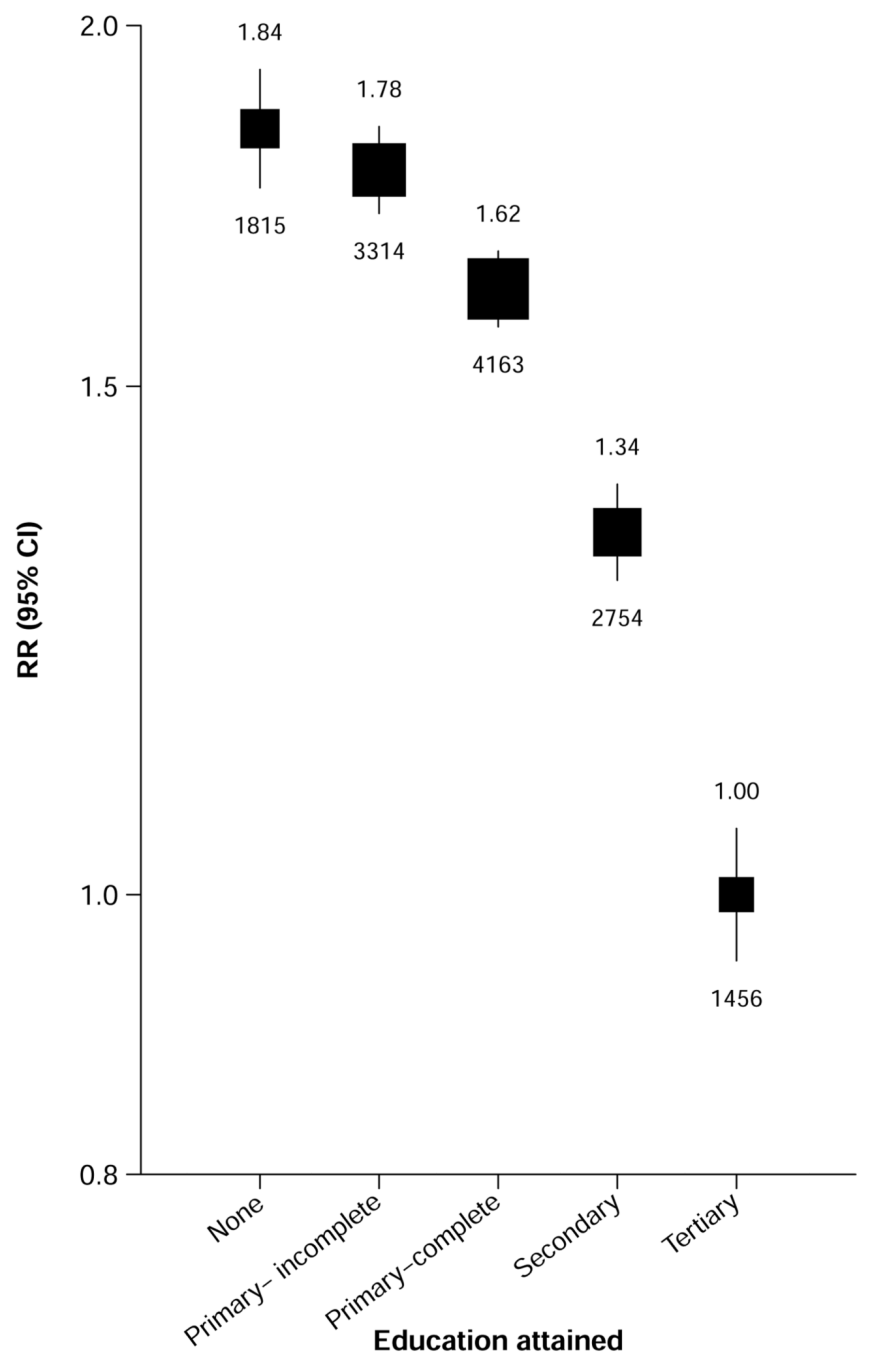
Relevance of education to mortality from any cause at ages 35-74 years Rate ratios (RRs) are stratified by age-at-risk and sex. The numbers above the squares are the RRs and the numbers below the squares are the number of deaths in that group. The size of each square is proportional to the amount of data available. The error bars represent 95% confidence intervals.

Education was strongly inversely associated with premature mortality; after adjustment for age and sex, participants with no education had almost twice the death rate of those with tertiary education (RR 1.84 [95% CI 1.71-1.98]) (Figure 2). The association appeared stronger in women (RR 2.03 [95% CI 1.87-2.21]) than in men (1.62 [1.45-1.81]) (Figure S1). Moreover, among women the inverse association was apparent at all levels of education (incomplete primary education: RR 1.93 [95% CI 1.85-2.02]; complete primary education: 1.68 [1.62-1.75]; secondary education: 1.39 [1.32-1.46]), while among men, death RRs were similar for those with no (RR 1.62 [95% CI 1.48-1.77]), incomplete primary (1.67 [1.58-1.77]) and complete primary (1.62 [1.55-1.70]) education. Sensitivity analyses showed no notable change in mortality RRs associated with education during the follow-up period (Table S4).

**Figure 3:**
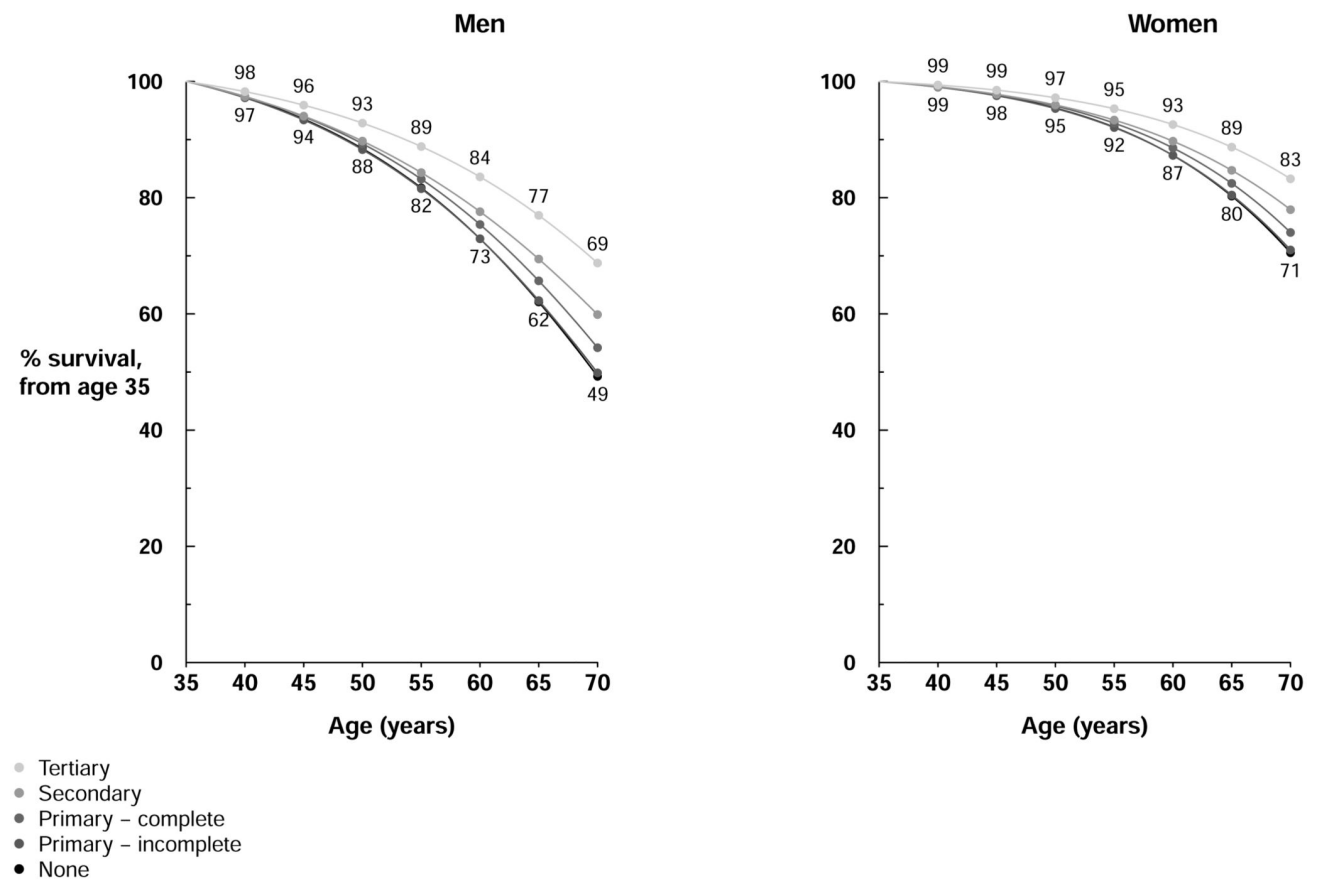
Hypothetical effect of mortality rate ratios associated with education on survival from age 35 to 70 years, at 2020 mortality rates in Mexico Absolute death rates at ages 35-70 years among people with different levels of education were estimated by combining age- and sex-specific 2020 national death rates in the eight component five-year age groups^22^ with study rate ratios (RRs) for all-cause mortality at ages 35-59 and 60-69 years, assuming 7%, 16%, 24%, 27%, and 25% of men had no education, incomplete primary education, complete primary education, secondary education and tertiary education, respectively, with corresponding proportions among women of 13%, 21%, 29%, 25% and 12%, respectively. For example, for each five year age group, the average annual mortality rate among men with tertiary education was estimated as A, and among men with other levels of education was estimated as the mortality RR for that level of education versus tertiary education multiplied by A, with A chosen such that (0.07 x RR_No education_ x A) + (0.16 x RR_Incomplete primary education_ x A) + (0.24 x RR_Complete primary education_ x A) + (0.27 x RR_Secondary education_ x A) + (0.25 x A) equalled the 2020 Mexican mortality rate for that five-year age group in men.

Applying our RRs for death from any cause to 2020 Mexican national death rates, Figure 3 shows estimated survival trajectories for men and women aged 35 to 70 by education. Among men, the 35-year probability of survival would be 69% in those with tertiary education compared with 49% in those with no formal education. Equivalent probabilities in women would be 83% and 71%, respectively. These correspond to approximately 6 years lower life expectancy from age 35 among men and women with no formal education when compared with those with tertiary education.

**Figure 4:**
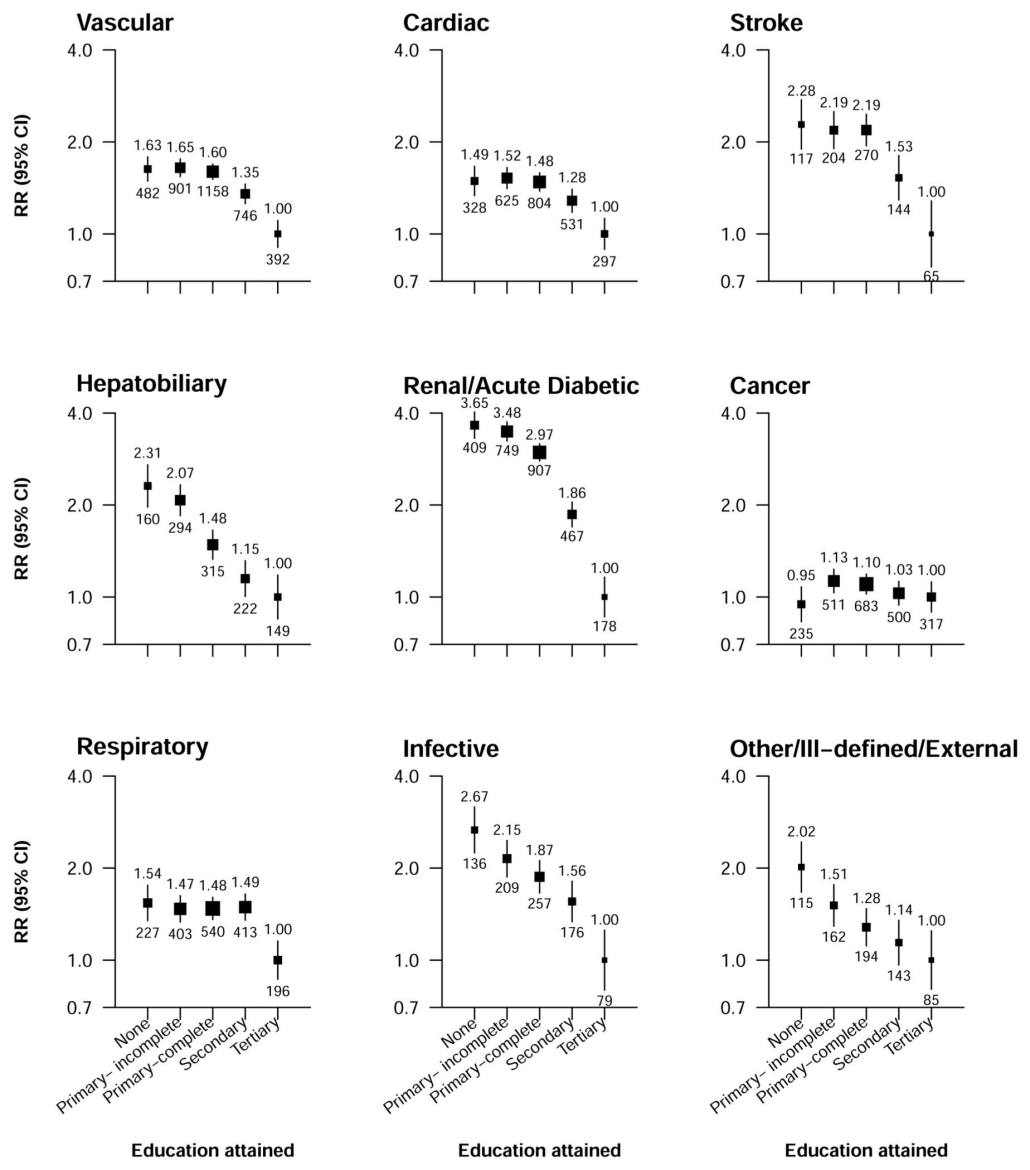
Relevance of education to cause specific mortality at ages 35-74 years Rate ratios (RRs) are stratified by age-at-risk and sex. The numbers above the squares are the RRs and the numbers below the squares are the number of deaths in that group. The size of each square is proportional to the amount of data available. The error bars represent 95% confidence intervals.

There was an inverse association of education with premature death from most, if not all, specific causes studied (Figure 4). RRs for death comparing those with no education versus those with tertiary education were highest for the composite of death from renal disease or an acute diabetic crisis (RR 3.65 [95% CI 3.05-4.38]), death from infectious disease (2.67 [2.00-3.56]) and, to a lesser extent, death from hepatobiliary diseases (2.31 [1.83-2.92]) and stroke (2.28 [1.67-3.12]). Death RRs for respiratory disease were lower only among participants with tertiary education, with no gradient in mortality rates at lower education levels (RRs between 1.47 [95% CI 1.23-1.75] and 1.54 [1.26-1.88], compared with tertiary education). There appeared to be only a weak inverse association with cancer mortality among participants who reported some education (RR 1.13 [95% CI 0.98-1.31] for incomplete primary versus tertiary education). The associations of education with death from specific causes were slightly stronger in women (Figure S2) than in men (Figure S3) with the exception of death from hepatobiliary disease, for which there was no apparent sex-difference. Education was less strongly associated with mortality at ages 75-84 than at 35–74 (Figure S4, Figure S5).

In exploratory mediation analyses, 84% of the association of education with mortality at ages 35-74 appeared to be attributable to lifestyle factors (smoking, alcohol consumption, leisure time physical activity), adiposity, diabetes status and SBP (Table S5). These same factors accounted for a similar proportion of the association with death from renal disease (79%) and from infectious disease (79%), moderately less—approximately 70%—of the association with death from vascular (69%) and hepatobiliary (74%) diseases, and a smaller proportion (51%) of the association with death from respiratory disease.

Among men, the associations of income with other baseline characteristics were broadly similar to those of education (Table S6), and premature mortality rates were higher at lower income levels (Figure S6). Men reporting no monthly income had more than twice the mortality rate of those reporting an income of ≥4500 pesos/month (RR 2.32 [95% CI 2.12-2.55]). Income was most strongly related to death from stroke (RR 2.89 [95% CI 1.96-4.26] for participants with no reported income versus ≥4500 pesos/month), hepatobiliary disease (RR 3.53 [2.63-4.73]) and renal disease/acute diabetic crises (RR 4.13 [3.30-5.17]) (Figure S7). Analyses examining the relevance of income for mortality among women are not presented since 64% reported no personal monthly income. Participants living in areas with a lower Social Development Index (indicating higher relative area-based deprivation) had higher death rates (Figure S8). The death RR comparing those living in areas in the bottom quintile of the Social Development Index with those living in areas in the top quintile was 1.58 (95% CI 1.49-1.67). The association was modestly weaker in men than in women (Figure S9). Inverse associations were also observed with death from most specific causes studied, with the exception of death from cancer for which there was no clear relationship (Figure S10).

## Discussion

In this study of Mexican adults we found marked social inequalities in mortality. Participants without formal education had premature death rates almost twice those of participants with tertiary education. Moreover, directionally consistent mortality gradients were observed with other socioeconomic exposures, including income (among men) and a population-specific social development index. Lifestyle and related physiological risk factors accounted for a large proportion of the observed educational inequalities in mortality.

Prior prospective evidence on the relevance of education for mortality in Mexico is limited to that from much smaller studies among older populations.^10–12^ The largest of these, based on data for approximately 14 000 participants in the Mexican Health and Aging Study (MHAS), showed 30% lower risk of mortality from any cause among participants with tertiary education when compared with those with no formal education after adjusting for age and sex.^11^ This is not inconsistent with our findings after taking account of the age (50+ years) of the MHAS population.^11^ However, reliance on this and other previously-published prospective evidence^10–12^ may underestimate the extent of educational inequalities in premature mortality. By contrast, we observed an approximately two-fold higher premature mortality rate among adults with no formal education, when compared with those with tertiary education, which is qualitatively and broadly quantitatively consistent with relationships reported across diverse populations.^2,7,24^

There were stronger inverse associations of education with death from acute renal and diabetic causes (which accounted for one in five premature deaths) and from infectious disease than with death from other specific causes in the present study. Higher diabetes prevalence among participants with lower education, coupled with notably high diabetes-associated mortality from renal disease and infectious disease^15^ in this population, likely contributed to these strong associations. Moreover, this appears consistent with previous evidence from Mexico, showing higher diabetes-related excess mortality in states with greater social disadvantage.^25^ Lower mortality with higher education was evident across all education levels for most causes of death. However, only adults with tertiary education had lower respiratory mortality, and education was only weakly inversely associated with cancer death. These findings contrast with those from previous studies, which have consistently reported inverse associations of education with respiratory^3,7^ and cancer^3,7,24^ mortality across all levels of education, possibly reflecting populations’ differing smoking patterns. There was no clear association of education with smoking among men in the Mexico City population, while more highly educated women were more likely to have smoked. This contrasts with a higher prevalence of smoking in lower socioeconomic groups observed in many high-, middle- and low-income settings,^26,27^ but is consistent with nationwide representative surveys in Mexico,^11,28^ and may reflect the relatively early stage of the tobacco epidemic in Mexico at the time of recruitment into the current study.^28^

We observed differences according to education level in the frequency of adverse lifestyle factors (with the exception of smoking among men) and in the levels of physiological correlates (including adiposity among women and blood pressure and diabetes in both sexes). Adjustment for these modifiable risk factors—whose associations with mortality have been described previously^15,29–31^—in exploratory mediation analyses suggested more than 80% of the association of education with premature mortality could be attributed to them. Many of these same factors have been found to partially account for the associations of socioeconomic position with mortality in other diverse populations,^7,32^ although direct comparisons between studies are complicated by differences in mediators explored and methodologies employed. In the present study, the mediators studied appeared to account for a greater proportion of the association of education with deaths from renal disease and from infectious disease than deaths from other causes, reflecting the greater relevance of diabetes to these causes in this population. Addressing social patterning of the lifestyle factors and physiological correlates studied (e.g., through fiscal interventions shown to reduce social inequalities in health outcomes^33^) could contribute significantly to reducing the observed inequalities and premature adult mortality more generally in this population. However, identification and effective mitigation of additional causes of inequalities will be important, perhaps particularly for deaths from, for example, respiratory and vascular diseases, for which these risk factors explained less of the association. Additional causes likely include the influence of education on knowledge-related assets, employment and, as shown in the present study, income and living conditions throughout the lifecourse, as well as social inequalities in access to effective healthcare.^34^ Some of these same factors have been proposed to underlie sex-differences in the association of education with mortality^4^ and may explain those observed in this population. However, determinants of social inequalities in health are complex. Although important, addressing mediating risk factors such as those studied herein is unlikely to be sufficient to eliminate health inequalities without accompanying efforts to address their social determinants.^35^

Investigation of the association of education and other measures of social position with mortality in a large single study with prolonged follow-up are major strengths of the presented analyses, enabling reliable estimates of educational and social inequalities in mortality in Mexico City. The focus on education reflects its relative ease of accurate measurement, its stability throughout adulthood (demonstrated by the consistency of self-reported education at baseline and resurvey in the present study), and its lesser susceptibility than other socioeconomic indicators to reverse causality within the context of mortality in middle-age. However, through additionally investigating income and the Social Development Index, we were able to demonstrate the consistent presence of social inequalities in mortality. The study also has limitations. Data were collected on individual income, which precluded assessment of the relevance of shared economic resources at the household level. As a consequence, we were unable to explore the association of income with mortality among women since almost two thirds reported none, which could reflect high or low socioeconomic position. This is consistent with the relatively high proportion of female participants who reported working in the household (approximately 70% at recruitment) and with described income patterns in Mexico.^36^ Inclusion of a health-related domain in the Social Development Index could lead to endogeneity bias in its association with mortality.^37^ However, qualitatively similar mortality gradients across quantiles of the Index’s six individual domains suggest any such bias was absent or negligible. Furthermore, we investigated the three socioeconomic indicators individually, but there may be value in studying them jointly to understand the complexity of pathways underlying inequalities in mortality. We were unable to assess all potential mediating factors, as described above, and were reliant on single baseline assessments of a few likely key mediators. Moreover, we could not account for unmeasured confounders influencing these associations. This might be expected to be particularly relevant for some specific causes of death studied (e.g., deaths from external causes). Moreover, we used single mediator measurements, which may have underestimated their contribution.^32^ Given these limitations, the presented mediation analyses should be considered largely exploratory. Misclassification of education level would also tend to lead to some underestimation of the strength of the relationship between education and mortality. Information on access to social security was available for only a subset of participants (since collection of these data commenced part way through recruitment); it was therefore not possible to reliably explore the relevance of social security for the observed social inequalities in mortality. The study population, from two municipalities of Mexico City, is not representative of the Mexican population. While studies aiming to estimate absolute risks require a representative population sample,^19,20^ the same is not generally true of studies attempting to characterise the *relative* risks of disease associated with exposures.^19,20^ Collider bias may affect studies of non-representative population samples in which the exposure and outcome, or other risk factors for the outcome, are associated with the probability of recruitment into the study.^19^ However, this would not be expected to explain the magnitude of associations observed in the present study,^19^ and large prospective studies of non-representative populations with heterogeneity of exposure status have been shown to provide reliable and generalisable evidence about the associations of risk factors with disease.^19–21^ By combining the generalisable education-associated mortality RRs from MCPS with national mortality data we derived estimates of absolute mortality risks from age 35 associated with lower educational attainment in the Mexican population, illustrating the potential public health relevance of the observed educational inequalities in mortality at a national level. However, these estimates do not take account of potential differences in causes of death between the study population and that of the whole of Mexico (e.g., those resulting from between-state differences in rates of homicide and femicide among adults in early middle-age^38^). Finally, the relatively small number of deaths from cancer—reflecting Mexico’s low cancer mortality rates^39^—precluded exploration of the association of education with cancer subtypes, of particular interest given the weak association observed when compared with other studies.^3,7,24^

In conclusion, in this large prospective study of Mexican adults, there were substantial social inequalities in mortality. When assessed through education, these accounted for an estimated 6-year gap in life expectancy between the most and least educated men and women. Known lifestyle and related physiological risk factors appeared to account for a large proportion of the observed mortality gradients, and effective population-level interventions to reduce these risk factors could lessen social inequalities in health and substantially reduce premature deaths among adults in Mexico City and in Mexico more generally.

## Supplementary Material

Supplementary Materials

## Figures and Tables

**Table 1:** Baseline characteristics of men and women aged 35-74 years by education at recruitment*

	Men (46 674)					Women (96 804)				
	None (3 390)	Primary - incomplete (7 529)	Primary - complete (11 403)	Secondary (12 633)	Tertiary (11 719)	None (12 358)	Primary - incomplete (20 083)	Primary - complete (28 257)	Secondary (24 313)	Tertiary (11 793)
Age, years †	60.7 (10.4)	57.8 (10.4)	53.1 (10.3)	47.5 (9.8)	46.9 (9.3)	58.8 (10.4)	54.8 (10.4)	50.1 (9.7)	45.5 (9.0)	44.6 (8.2)
Income, pesos/month	1306 (2065)	1723 (1663)	2358 (2900)	3195 (3712)	6679 (8747)	288 (673)	366 (911)	545 (1272)	1075 (2039)	3166 (4842)
Social Development Index ††	0.72 (0.08)	0.73 (0.09)	0.74 (0.10)	0.76 (0.10)	0.81 (0.11)	0.72 (0.09)	0.73 (0.10)	0.75 (0.10)	0.77 (0.11)	0.82 (0.11)
Smoking status										
Current smoker	1488 (44%)	3631 (48%)	5939 (52%)	7172 (57%)	5564 (47%)	1503 (12%)	3378 (17%)	6693 (24%)	7701 (32%)	3811 (32%)
Ex-smoker	1195 (35%)	2619 (35%)	3341 (29%)	3162 (25%)	3160 (27%)	1565 (13%)	2613 (13%)	3886 (14%)	3679 (15%)	2186 (19%)
Never smoker	707 (21%)	1279 (17%)	2123 (19%)	2299 (18%)	2995 (26%)	9290 (75%)	14 092 (70%)	17 678 (63%)	12 933 (53%)	5796 (49%)
Drinking behaviour										
Current drinker	2162 (64%)	5366 (71%)	8698 (76%)	10 129 (80%)	9862 (84%)	5930 (48%)	11 571 (58%)	18 051 (64%)	16 755 (69%)	8595 (73%)
Former drinker	931 (27%)	1750 (23%)	2070 (18%)	1844 (15%)	1112 (9%)	2033 (16%)	2786 (14%)	3190 (11%)	2277 (9%)	867 (7%)
Never drinker	297 (9%)	413 (5%)	635 (6%)	660 (5%)	745 (6%)	4395 (36%)	5726 (29%)	7016 (25%)	5281 (22%)	2331 (20%)
Leisure-time physical activity										
None	2895 (85%)	6043 (80%)	8353 (73%)	8416 (67%)	6826 (58%)	11 148 (90%)	17 459 (87%)	23 319 (83%)	18 685 (77%)	7980 (68%)
Up to 2 times/week	175 (5%)	618 (8%)	1405 (12%)	2136 (17%)	2046 (17%)	473 (4%)	872 (4%)	1284 (5%)	1423 (6%)	992 (8%)
Regular, ≥3 times/week	320 (9%)	868 (12%)	1645 (14%)	2081 (16%)	2847 (24%)	737 (6%)	1752 (9%)	3654 (13%)	4205 (17%)	2821 (24%)
Anthopometry, blood pressure and HbA1c										
Body mass index, kg/m^2^	27.7 (4.3)	28.2 (4.2)	28.2 (4.2)	28.1 (4.2)	27.8 (4.1)	30.2 (5.3)	30.4 (5.2)	30.0 (5.1)	29.1 (5.0)	28.0 (4.9)
Waist circumference, cm	97 (10)	97 (10)	96 (10)	96 (10)	96 (10)	97 (12)	96 (12)	94 (12)	91 (12)	89 (12)
Hip circumference, cm	100 (8)	101 (8)	101 (8)	101 (8)	102 (8)	107 (12)	107 (11)	107 (11)	106 (11)	104 (11)
Systolic blood pressure, mmHg	133 (17)	132 (17)	129 (16)	126 (14)	126 (14)	134 (19)	130 (17)	126 (16)	122 (15)	120 (14)
Diastolic blood pressure, mmHg	85 (10)	85 (10)	85 (10)	84 (10)	84 (9)	85 (11)	84 (10)	83 (10)	81 (10)	80 (9)
HbA1c, % $	5.8 (1.1)	5.8 (1.1)	5.7 (1.0)	5.6 (1.0)	5.5 (0.8)	5.9 (1.2)	5.8 (1.1)	5.7 (1.0)	5.5 (0.8)	5.4 (0.7)
Prior diseases ||										
Diabetes	643 (19%)	1414 (19%)	1807 (16%)	1285 (10%)	940 (8%)	2827 (23%)	3814 (19%)	3611 (13%)	1637 (7%)	528 (4%)
Cardiovascular disease	116 (3%)	288 (4%)	353 (3%)	257 (2%)	248 (2%)	354 (3%)	487 (2%)	554 (2%)	335 (1%)	152 (1%)
Cancer	14 (<0.5%)	38 (1%)	72 (1%)	54 (<0.5%)	67 (1%)	179 (1%)	302 (2%)	407 (1%)	348 (1%)	188 (2%)
Other #	182 (5%)	450 (6%)	613 (5%)	536 (4%)	547 (5%)	1160 (9%)	2090 (10%)	2948 (10%)	2153 (9%)	1074 (9%)

Results shown are frequencies (column percentages) or mean (standard deviation); HbA1c = haemoglobin A1c.

* n=143 478 from an initial sample of 159 517 after excluding those with missing values of education, smoking, physical activity, alcohol, height, weight, waist circumference, hip circumference, waist-to-hip ratio and blood pressure (n=2597), those with height<120cm, or height>200cm or weight<35kg or weight>250kg or BMI<15kg/m^2^ or BMI>60kg/m^2^ or waist circumference<60cm or waist circumference>180cm or hip circumference<70cm or hip circumference>180cm or waist-to-hip ratio<0.5 or waist-to-hip ratio>1.5 (n=629), those with uncertain cause of death (n=1393) and those with age-at-risk≥75 years (n=13 367)

† Median (interquartile range): 50.5 years (41.5, 59.5) for men and 48.5 years (41.5, 58.5) for women.

†† Higher scores represent higher area-based social development.

$ HbA1c among participants without previously-diagnosed diabetes.

|| Self-reported diseases.

# Other diseases include emphysema, chronic kidney disease, peptic ulcer, cirrhosis, and peripheral arterial disease.

## Data Availability

Data from the Mexico City Prospective Study are available to *bona fide* researchers. For more details, the study’s Data and Sample Sharing policy may be downloaded (in English or Spanish) from https://www.ctsu.ox.ac.uk/research/mcps. Available study data can be examined in detail through the study’s Data Showcase, available at https://datashare.ndph.ox.ac.uk/mexico/.

## Research in context

### Evidence before this study

We undertook a literature search in PubMed to identify articles published in English before 23 June 2023 that reported on prospective studies examining the association of education, or other indicators of socioeconomic position, with mortality in a Mexican or Latin American population, using the terms (“socioeconomic” OR “socio-economic” OR “education”) AND (“mortality” OR “death”) AND (“Mexico” OR “Mexican” OR “Latin America” OR “Latin American”). Reference lists of relevant articles were also reviewed. There were few prospective studies examining social inequalities in mortality among adults in Mexico. The largest involved fewer than 14 000 participants and investigated only overall mortality. None investigated factors mediating the association of education with mortality. One large prospective Latin American population-based study was identified, including approximately 150 000 adults in Cuba. This found a strong inverse association between education and death, one-third of which was explained by lifestyle factors in men and one-fifth in women. However, generalisability of these findings to other countries in the region with very different social and economic contexts is unclear.

### Added value of this study

The present study, including approximately 150 000 men and women from Mexico City, showed strong social, including educational, inequalities in premature adult mortality. There was an inverse association of education with overall adult mortality; given age and sex, the death RR at ages 35-74 years for participants with no education, when compared with participants with tertiary education, was 1.84. Deaths from renal disease and acute diabetic crises (RR 3.65) and from infectious disease (RR 2.67) were most strongly associated with education, but higher education was associated with lower risk of death from most, if not all, specific causes. Income among men and an area-based measure of social development—the Social Development Index—were also inversely associated with overall and cause-specific mortality. Lifestyle factors (smoking, alcohol consumption and leisure time physical activity) and their physiological correlates (adiposity, diabetes and blood pressure) appeared to account for a large proportion (approximately 80%) of the association of education with death. Applying our RRs for overall mortality to 2020 Mexican national death rates, men and women with no formal education were estimated to have approximately 6 years lower life expectancy from age 35 than those with tertiary education.

### Implications of all the available evidence

There are marked social inequalities in mortality among adults in Mexico City. Addressing these major behavioural risk factors and their associated physiological correlates would be expected to contribute to reducing these disparities in health and, in turn, to reducing premature adult mortality in this population.
