# Supplementary material for: Educational and social inequalities and cause-specific mortality: a prospective study in two municipalities of Mexico City
Source: Lancet Public Health. Author manuscript; Available in PMC 2023 Nov 1. (PMC7615266; doi:10.1016/S2468-2667(23)00153-6)
Supplement: Supplementary Materials [file EMS181802-supplement-Supplementary_Materials.pdf]

Educational and social inequalities and cause-specific mortality: a prospective study in two municipalities of Mexico City

Online Supplemental Material, Table of Contents

**Supplementary tables**	**Page**
Table S1. Number of deaths at ages 35-74 years, by underlying cause (ICD-10 code)	2
Table S2. Baseline characteristics of residents of Coyoacán and Iztapalapa aged 35-74 years by education at recruitment	4
Table S3. Education reported at baseline and resurvey among 2971 men and 6970 women resurveyed 16 years later	5
Table S4. Relevance of education to mortality from any cause at ages 35−74 years during the first 10 years of follow-up vs. subsequent years combined	6
Table S5. Associations of education with all-cause and cause-specific mortality after adjustment for potential mediators	7
Table S6. Baseline characteristics of 46 674 men aged 35-74 years by reported income at recruitment	8
**Supplementary figures**	
Figure S1. Relevance of education to mortality from any cause at ages 35−74 years, by sex	9
Figure S2. Relevance of education to cause−specific mortality among women at ages 35−74 years	10
Figure S3. Relevance of education to cause−specific mortality among men at ages 35−74 years	11
Figure S4. Relevance of education to mortality from any cause at ages 75−84 years	12
Figure S5. Relevance of education to cause−specific mortality at ages 75−84 years	13
Figure S6. Relevance of income to mortality from any cause among men at ages 35−74 years	14
Figure S7. Relevance of income to cause−specific mortality among men at ages 35−74 years	15
Figure S8. Relevance of Social Development Index to mortality from any cause at ages 35−74 years	16
Figure S9. Relevance of Social Development Index to mortality from any cause at ages 35−74 years, by sex	17
Figure S10. Relevance of Social Development Index to cause−specific mortality at ages 35−74 years	18

Table S1. Number of deaths at ages 35-74 years, by underlying cause (ICD-10 code)

Underlying cause of death	ICD-10 codes (and number of deaths)
CARDIAC (n=2585)	I011 (1), I018 (1), I050 (3), I051 (2), I059 (22), I069 (2), I070 (1), I071 (2), I079 (2), I080 (3), I081 (1), I091 (1), I099 (17), I110 (110), I119 (10), I200 (4), I209 (3), I210 (21), I211 (12), I213 (1), I219 (1870), I220 (1), I221 (1), I249 (12), I251 (50), I252 (1), I255 (1), I258 (9), I259 (97), I270 (7), I272 (2), I279 (8), I301 (1), I319 (1), I330 (6), I340 (4), I348 (1), I350 (12), I351 (1), I358 (2), I38X (9), I420 (12), I421 (1), I426 (2), I429 (2), I442 (11), I443 (1), I460 (1), I469 (10), I471 (4), I472 (3), I480 (1), I489 (4), I48X (8), I490 (7), I499 (5), I500 (57), I501 (14), I509 (78), I515 (2), I518 (4), I519 (11), Q210 (1), Q213 (1), Q231 (1), Q238 (1), Q248 (1), R570 (27)
STROKE (n=800)	F019 (2), I600 (2), I602 (1), I608 (2), I609 (80), I61 (1), I610 (1), I612 (2), I613 (1), I614 (1), I615 (2), I618 (1), I619 (243), I620 (9), I629 (4), I633 (4), I634 (17), I635 (3), I638 (2), I639 (60), I64X (103), I669 (6), I671 (6), I672 (2), I673 (1), I674 (4), I678 (79), I679 (120), I690 (2), I691 (1), I693 (7), I694 (5), I698 (26)
OTHER VASCULAR (n=294)	E115 (37), E145 (37), I260 (2), I269 (75), I710 (4), I712 (1), I713 (4), I714 (3), I718 (3), I719 (1), I729 (1), I731 (1), I739 (3), I740 (1), I741 (1), I771 (17), I776 (1), I779 (1), I802 (6), I803 (1), I822 (1), I828 (1), I829 (3), I830 (1), I839 (1), I872 (5), I879 (1), I890 (1), I99X (2), K550 (67), K551 (1), K552 (1), K559 (8), K761 (1)
RENAL (n=2153)	E102 (11), E112 (911), E122 (1), E142 (376), I120 (109), I129 (2), I130 (3), I131 (2), I132 (29), N002 (1), N009 (2), N039 (28), N049 (2), N059 (10), N10X (2), N119 (1), N12X (8), N142 (1), N151 (12), N170 (1), N179 (82), N180 (12), N185 (30), N189 (219), N19X (59), N200 (7), N201 (1), N281 (1), N289 (3), N300 (1), N309 (1), N390 (223), Q619 (1), Y841 (1)
ACUTE DIABETIC (n=557)	E100 (3), E101 (3), E110 (174), E111 (156), E121 (1), E140 (117), E141 (97), E162 (6)
HEPATOBILIARY (n=1140)	B169 (5), B171 (31), B181 (2), B182 (8), B189 (1), B190 (2), B199 (2), D136 (1), I850 (17), I859 (5), K563 (1), K701 (20), K702 (1), K703 (170), K704 (24), K709 (18), K711 (2), K716 (1), K720 (9), K721 (65), K729 (204), K739 (2), K742 (1), K743 (3), K745 (2), K746 (309), K750 (14), K754 (3), K759 (2), K760 (1), K764 (1), K766 (14), K767 (15), K768 (1), K769 (17), K800 (5), K801 (9), K802 (3), K803 (4), K804 (1), K805 (3), K810 (17), K811 (4), K819 (6), K821 (1), K822 (1), K829 (6), K830 (19), K831 (3), K851 (1), K852 (4), K858 (7), K859 (37), K85X (28), K861 (3), K868 (3), Q447 (1)
CANCER (n=2246)	C029 (10), C049 (1), C069 (2), C07X (1), C089 (1), C109 (3), C119 (3), C139 (1), C140 (5), C142 (1), C159 (13), C160 (2), C169 (209), C170 (10), C179 (1), C182 (2), C184 (1), C187 (2), C189 (125), C19X (4), C20X (20), C220 (55), C221 (22), C229 (116), C23X (29), C240 (11), C241 (9), C248 (3), C249 (14), C250 (24), C252 (1), C259 (99), C260 (3), C269 (2), C319 (1), C329 (16), C33X (1), C349 (166), C37X (1), C382 (1), C383 (1), C384 (1), C402 (1), C411 (1), C412 (2), C414 (1), C419 (7), C435 (1), C437 (1), C438 (1), C439 (15), C444 (1), C445 (1), C447 (1), C449 (6), C450 (1), C451 (2), C457 (1), C459 (3), C469 (1), C479 (1), C480 (10), C482 (5), C490 (1), C492 (4), C495 (2), C499 (13), C509 (239), C519 (4), C530 (2), C539 (134), C541 (22), C549 (2), C55X (12), C56X (110), C609 (2), C61X (75), C629 (1), C64X (91), C66X (1), C679 (24), C680 (3), C689 (1), C709 (2), C710 (16), C711 (1), C718 (2), C719 (41), C720 (1), C729 (1), C73X (27), C741 (1), C749 (1), C759 (1), C760 (6), C761 (1), C762 (2), C763 (2), C764 (1), C765 (2), C779 (1), C780 (8), C786 (3), C787 (8), C788 (2), C793 (3), C794 (3), C795 (1), C796 (1), C798 (3), C800 (29), C809 (16), C80X (4), C817 (1), C819 (10), C822 (1), C829 (1), C833 (12), C838 (1), C839 (3), C844 (1), C845 (3), C851 (1), C857 (2), C859 (49), C900 (43), C901 (1), C902 (1), C910 (24), C911 (1), C919 (2), C920 (29), C921 (8), C927 (3), C929 (4), C950 (1), C959 (3), C97X (1), D371 (3), D372 (1), D374 (4), D376 (6), D377 (1), D380 (2), D381 (4), D383 (2), D391 (2), D397 (1), D410 (2), D419 (1), D430 (11), D432 (1), D449 (1), D486 (1), D487 (5), D489 (3)
RESPIRATORY (n=1779)	A162 (9), A165 (1), A169 (2), B206 (2), B440 (1), B441 (1), B909 (1), E848 (1), J069 (1), J09 (3), J100 (2), J111 (1), J129 (4), J151 (3), J157 (1), J159 (33), J180 (60), J181 (34), J182 (2), J188 (1), J189 (610), J209 (4), J22X (23), J348 (1), J391 (1), J42X (17), J439 (34), J440 (104), J441 (2), J448 (7), J449 (166), J459 (12), J46X (2), J47X (2), J60X (1), J64X (5), J677 (1), J679 (2), J680 (1), J690 (3), J80X (2), J81X (6), J841 (82), J848 (1), J849 (11), J850 (1), J852 (1), J869 (8), J90X (5), J920 (1), J939 (1), J948 (1), J960 (3), J961 (1), J969 (4), J980 (1), J981 (1), J984 (14), J985 (4), J988 (8), J989 (1), Q311 (1), U071 (240), U072 (220)
INFECTIVE (n=857)	A047 (3), A060 (1), A090 (18), A099 (31), A09X (12), A170 (2), A180 (1), A181 (2), A182 (1), A183 (1), A199 (3), A415 (1), A419 (214), A480 (1), A483 (1), A498 (1), A810 (2), A86X (2), B200 (1), B201 (2), B207 (3), B208 (9), B210 (2), B212 (1), B218 (1), B227 (2), B238 (6), B24X (6), B373 (1), B465 (2), B690 (2), B948 (2), B99X (1), G009 (3), G039 (4), G042 (1), G049 (7), G060 (3), H440 (1), H664 (1), K052 (1), K222 (1), K223 (2), K228 (1), K251 (3), K254 (11), K255 (12), K256 (5), K259 (6), K264 (6), K265 (3), K269 (2), K274 (3), K275 (1), K290 (6), K291 (2), K292 (1), K295 (4), K297 (1), K318 (6), K319 (1), K352 (4), K353 (4), K358 (4), K359 (2), K572 (2), K578 (8), K579 (12), K610 (4), K611 (1), K612 (1), K650 (22), K658 (2), K659 (61), K920 (27), K921 (2), K922 (126), L021 (3), L022 (6), L023 (2), L024 (2), L031 (4), L038 (1), L039 (4), L089 (36), L899 (6), L89X (5), L905 (1), L984 (7), M009 (2), M600 (2), M725 (1), M726 (16), M798 (25), M869 (3), N410 (1), N498 (7), N499 (1), N719 (1), N739 (2), N764 (1)
OTHER DEFINED MEDICAL (n=400)	D033 (1), D27X (1), D329 (9), D352 (1), D464 (1), D467 (1), D469 (6), D479 (1), D619 (7), D649 (4), D65X (1), D682 (1), D693 (2), D694 (1), D696 (3), D699 (3), D70X (2), D733 (1), D739 (1), D762 (1), E031 (1), E035 (1), E039 (16), E049 (1), E055 (1), E059 (3), E065 (1), E116 (3), E119 (6), E129 (1), E146 (4), E149 (7), E230 (1), E249 (2), E279 (1), E43X (1), E440 (1), E660 (1), E725 (1), E835 (1), E875 (1), E876 (1), E878 (1), E889 (2), F03X (5), F09X (1), F102 (7), F182 (1), F209 (1), G10X (6), G121 (1), G122 (18), G20X (9), G300 (1), G309 (5), G310 (1), G35X (2), G379 (1), G403 (1), G409 (12), G419 (1), G439 (1), G589 (1), G610 (5), G709 (1), G710 (2), G809 (1), G822 (1), G919 (4), G931 (12), G934 (6), G935 (1), G936 (2), G938 (1), G958 (1), G959 (1), I10X (2), K088 (1), K102 (1), K137 (1), K389 (1), K404 (1), K419 (1), K420 (4), K421 (1), K429 (1), K430 (3), K439 (2), K440 (1), K460 (2), K461 (1), K469 (3), K513 (1), K519 (1), K529 (6), K560 (1), K562 (1), K566 (36), K567 (1), K593 (5), K628 (1), K630 (1), K631 (22), K632 (4), K635 (1), K638 (2), K639 (5), K661 (1), K918 (1), K931 (1), L100 (1), L109 (1), L511 (1), L512 (2), L921 (1), L958 (1), L988 (1), M050 (1), M068 (1), M069 (16), M100 (1), M109 (1), M139 (1), M311 (2), M313 (1), M319 (1), M321 (4), M329 (3), M331 (1), M340 (1), M348 (1), M349 (1), M623 (4), M799 (1), M844 (1), M993 (1), N40X (8), N939 (1), N948 (1), O622 (1), O720 (1)

Table S1. Number of deaths at ages 35-74 years, by underlying cause (ICD-10 code)

Underlying cause of death	ICD-10 codes (and number of deaths)
EXTERNAL (n=392)	S729 (2), T07X (1), V011 (1), V029 (1), V041 (1), V049 (1), V051 (1), V059 (1), V093 (3), V099 (75), V182 (1), V209 (1), V299 (1), V439 (1), V489 (1), V494 (1), V496 (1), V499 (13), V580 (1), V719 (1), V785 (1), V878 (6), V892 (9), V899 (5), W018 (1), W060 (1), W100 (15), W104 (1), W105 (1), W108 (2), W126 (1), W130 (11), W134 (3), W135 (1), W138 (1), W139 (2), W170 (4), W172 (1), W174 (1), W176 (1), W178 (2), W179 (1), W18 (1), W180 (5), W181 (1), W184 (3), W188 (1), W190 (11), W194 (1), W195 (1), W199 (3), W200 (1), W206 (1), W250 (1), W314 (1), W340 (1), W370 (1), W704 (1), W744 (2), W748 (2), W769 (1), W780 (1), W789 (1), W799 (1), W849 (2), W878 (1), X09 (1), X090 (3), X094 (1), X219 (1), X314 (1), X360 (1), X459 (1), X470 (1), X590 (4), X594 (1), X598 (1), X599 (30), X640 (1), X650 (1), X680 (1), X700 (6), X702 (1), X708 (1), X740 (3), X780 (3), X800 (1), X910 (1), X912 (1), X914 (2), X950 (3), X954 (18), X955 (1), X959 (1), X990 (8), X994 (6), X999 (2), Y044 (2), Y048 (1), Y084 (1), Y094 (1), Y099 (1), Y159 (1), Y200 (1), Y240 (1), Y244 (3), Y245 (1), Y248 (1), Y249 (1), Y260 (2), Y280 (1), Y330 (1), Y334 (1), Y338 (1), Y340 (5), Y344 (4), Y346 (1), Y348 (2), Y349 (15), Y405 (1), Y579 (3), Y835 (1), Y838 (1), Y839 (5), Y846 (1)
UNDEFINED (n=299)	E86X (4), E870 (1), E872 (9), E874 (1), R040 (1), R100 (2), R11X (1), R190 (1), R571 (13), R579 (3), R58X (3), R64X (1), R688 (20), R69X (1), R99X (238)

Table S2. Baseline characteristics of residents of Coyoacán and Iztapalapa aged 35-74 years by education at recruitment*

	Coyoacán (58 301)					Iztapalapa (85 177)				
	**None (5 816)**	**Primary - incomplete (9 909)**	**Primary - complete (15 097)**	**Secondary (14 896)**	**Tertiary (12 583)**	**None (9 932)**	**Primary - incomplete (17 703)**	**Primary - complete (24 563)**	**Secondary (22 050)**	**Tertiary (10 929)**
Age, years †	59.3 (10.0)	55.6 (10.2)	51.5 (9.8)	47.0 (9.4)	46.5 (9.0)	59.2 (10.7)	55.7 (10.6)	50.7 (10.0)	45.6 (9.2)	44.9 (8.6)
Income, pesos/month	548 (972)	790 (1251)	1146 (2012)	2009 (3156)	5805 (8058)	483 (1320)	706 (1344)	1017 (2088)	1659 (2719)	3893 (6105)
Social Development Index ††	0.75 (0.08)	0.77 (0.10)	0.80 (0.11)	0.82 (0.11)	0.86 (0.11)	0.70 (0.09)	0.71 (0.09)	0.72 (0.09)	0.74 (0.09)	0.76 (0.09)
Social security system use $										
Social security						3395 (34%)	6707 (38%)	9461 (39%)	8391 (38%)	4682 (43%)
Other public services						2865 (29%)	4552 (26%)	5673 (23%)	4700 (21%)	1269 (12%)
Private/Other						2622 (26%)	4461 (25%)	6305 (26%)	6064 (28%)	3046 (28%)
Smoking status										
Current smoker	1222 (21%)	2664 (27%)	5083 (34%)	6421 (43%)	5249 (42%)	1769 (18%)	4345 (25%)	7549 (31%)	8452 (38%)	4126 (38%)
Ex-smoker	1077 (19%)	1944 (20%)	2865 (19%)	2870 (19%)	2982 (24%)	1683 (17%)	3288 (19%)	4362 (18%)	3971 (18%)	2364 (22%)
Never smoker	3517 (60%)	5301 (53%)	7149 (47%)	5605 (38%)	4352 (35%)	6480 (65%)	10 070 (57%)	12 652 (52%)	9627 (44%)	4439 (41%)
Drinking behaviour										
Current drinker	3230 (56%)	6388 (64%)	10 722 (71%)	11 447 (77%)	10 340 (82%)	4862 (49%)	10 549 (60%)	16 027 (65%)	15 437 (70%)	8117 (74%)
Former drinker	1137 (20%)	1628 (16%)	1968 (13%)	1579 (11%)	988 (8%)	1827 (18%)	2908 (16%)	3292 (13%)	2542 (12%)	991 (9%)
Never drinker	1449 (25%)	1893 (19%)	2407 (16%)	1870 (13%)	1255 (10%)	3243 (33%)	4246 (24%)	5244 (21%)	4071 (18%)	1821 (17%)
Leisure-time physical activity										
None	5156 (89%)	8353 (84%)	11 570 (77%)	10 266 (69%)	7413 (59%)	8887 (89%)	15 149 (86%)	20 102 (82%)	16 835 (76%)	7393 (68%)
Up to 2 times/week	221 (4%)	510 (5%)	1072 (7%)	1469 (10%)	1732 (14%)	427 (4%)	980 (6%)	1617 (7%)	2090 (9%)	1306 (12%)
Regular, ≥3 times/week	439 (8%)	1046 (11%)	2455 (16%)	3161 (21%)	3438 (27%)	618 (6%)	1574 (9%)	2844 (12%)	3125 (14%)	2230 (20%)
Anthopometry, blood pressure and HbA1c										
Body mass index, kg/m²	29.3 (5.1)	29.4 (5.0)	29.0 (4.9)	28.2 (4.6)	27.4 (4.3)	29.9 (5.2)	30.0 (5.1)	29.8 (5.0)	29.1 (4.9)	28.5 (4.6)
Waist circumference, cm	96 (11)	95 (11)	93 (11)	91 (11)	91 (11)	97 (11)	97 (11)	95 (11)	94 (12)	93 (12)
Hip circumference, cm	105 (11)	105 (11)	105 (11)	103 (10)	103 (9)	106 (11)	106 (11)	106 (11)	105 (11)	104 (10)
Systolic blood pressure, mmHg	136 (19)	132 (17)	129 (16)	125 (15)	124 (14)	132 (18)	130 (17)	126 (16)	122 (15)	122 (14)
Diastolic blood pressure, mmHg	86 (11)	86 (10)	84 (10)	83 (10)	83 (10)	85 (10)	84 (10)	83 (10)	81 (10)	81 (9)
HbA1c, % ||	5.7 (1.1)	5.7 (1.1)	5.5 (1.0)	5.4 (0.8)	5.4 (0.7)	5.9 (1.2)	5.9 (1.1)	5.7 (1.0)	5.6 (0.9)	5.6 (0.8)
Prior diseases #										
Diabetes	1146 (20%)	1721 (17%)	1859 (12%)	1066 (7%)	724 (6%)	2324 (23%)	3507 (20%)	3559 (14%)	1856 (8%)	744 (7%)
Cardiovascular disease	222 (4%)	332 (3%)	436 (3%)	321 (2%)	245 (2%)	248 (2%)	443 (3%)	471 (2%)	271 (1%)	155 (1%)
Cancer	87 (1%)	152 (2%)	231 (2%)	194 (1%)	166 (1%)	106 (1%)	188 (1%)	248 (1%)	208 (1%)	89 (1%)
Other **	719 (12%)	1268 (13%)	1977 (13%)	1581 (11%)	1101 (9%)	623 (6%)	1272 (7%)	1584 (6%)	1108 (5%)	520 (5%)

Results shown are frequencies (column percentages) or mean (standard deviation); HbA1c = haemoglobin A1c.
* Same exclusions applied as described in Table 1
† Median (interquartile range): 49.5 years (41.5, 58.5) for residents of Coyoacán, 48.5 years (41.5, 58.5) for residents of Iztapalapa
†† Higher scores represent higher area-based social development.
$ Collection of data on social security was commenced part way through recruitement. The percentages shown relate to participants who were asked this question, all of whom resided in Iztapalapa.
|| HbA1c among participants without previously-diagnosed diabetes.
Self-reported diseases.
** Other diseases include emphysema, chronic kidney disease, peptic ulcer, cirrhosis, and peripheral arterial disease.

Table S3. Education reported at baseline and resurvey among 2971 men and 6970 women resurveyed 16 years later*

	Reported at resurvey					
Reported at baseline	None (1 358)	Primary - incomplete (2 214)	Primary - complete (2 701)	Secondary (3 001)	Tertiary (667)	Total (9 941)
None	882 (65%)	204 (9%)	44 (2%)	9 (<0.5%)	6 (1%)	1145 (12%)
Primary - incomplete	371 (27%)	1602 (72%)	224 (8%)	50 (2%)	6 (1%)	2253 (23%)
Primary - complete	68 (5%)	363 (16%)	2192 (81%)	404 (13%)	23 (3%)	3050 (31%)
Secondary	21 (2%)	38 (2%)	212 (8%)	1966 (66%)	89 (13%)	2326 (23%)
Tertiary	16 (1%)	7 (<0.5%)	29 (1%)	572 (19%)	543 (81%)	1167 (12%)

*Kappa=0.64. This describes the agreement between recruitment and resurvey values.
Frequencies and column percentages are presented.

Table S4. Relevance of education to mortality from any cause at ages 35-74 years during the first 10 years of follow-up vs. subsequent years of follow-up combined

Education attained	First 10 years of follow-up		Subsequent follow-up	
	Deaths	HR (95% CI)	Deaths	HR (95% CI)
None	1206	1.76 (1.66, 1.86)	609	1.77 (1.63, 1.92)
Primary-incomplete	1986	1.71 (1.64, 1.79)	1328	1.75 (1.66, 1.85)
Primary-complete	2093	1.53 (1.47, 1.60)	2070	1.67 (1.60, 1.75)
Secondary	1247	1.28 (1.21, 1.35)	1507	1.38 (1.31, 1.45)
Tertiary	658	1.00 (0.92, 1.08)	798	1.00 (0.93, 1.07)

Table S5. Associations of education with all-cause and cause-specific mortality after adjustment for potential mediators

Mortality causes	Education attained	Basic adjustment*		Adjusted for measured mediators †		%reduction	
		RR (95% CI)	x^2 ††	RR (95% CI)	x^2 $	x^2	Log RR
All-cause	Tertiary	1.00 (0.95, 1.05)	446.1	1.00 (0.95, 1.06)	70.1	84	74
	Secondary	1.34 (1.28, 1.39)		1.20 (1.15, 1.25)			
	Primary - Complete	1.62 (1.57, 1.67)		1.28 (1.25, 1.32)			
	Primary - Incomplete	1.78 (1.72, 1.85)		1.25 (1.21, 1.30)			
	None	1.84 (1.76, 1.93)		1.17 (1.12, 1.23)			
Vascular	Tertiary	1.00 (0.90, 1.11)	87.1	1.00 (0.90, 1.11)	26.9	69	102
	Secondary	1.35 (1.26, 1.46)		1.20 (1.11, 1.29)			
	Primary - Complete	1.60 (1.51, 1.69)		1.24 (1.17, 1.32)			
	Primary - Incomplete	1.65 (1.54, 1.76)		1.12 (1.05, 1.20)			
	None	1.63 (1.49, 1.79)		0.99 (0.90, 1.09)			
Cardiac	Tertiary	1.00 (0.89, 1.12)	44.4	1.00 (0.89, 1.13)	14.3	68	118
	Secondary	1.28 (1.18, 1.40)		1.14 (1.04, 1.25)			
	Primary - Complete	1.48 (1.38, 1.58)		1.16 (1.08, 1.24)			
	Primary - Incomplete	1.52 (1.41, 1.65)		1.06 (0.98, 1.15)			
	None	1.49 (1.33, 1.66)		0.93 (0.83, 1.04)			
Stroke	Tertiary	1.00 (0.78, 1.28)	48.3	1.00 (0.77, 1.29)	15.7	67	74
	Secondary	1.53 (1.29, 1.80)		1.33 (1.12, 1.57)			
	Primary - Complete	2.19 (1.94, 2.46)		1.63 (1.45, 1.83)			
	Primary - Incomplete	2.19 (1.90, 2.51)		1.38 (1.20, 1.59)			
	None	2.28 (1.89, 2.75)		1.24 (1.02, 1.50)			
Hepatobiliary	Tertiary	1.00 (0.85, 1.18)	86.6	1.00 (0.84, 1.19)	22.8	74	51
	Secondary	1.15 (1.00, 1.31)		1.04 (0.91, 1.19)			
	Primary - Complete	1.48 (1.33, 1.65)		1.21 (1.08, 1.35)			
	Primary - Incomplete	2.07 (1.85, 2.33)		1.48 (1.32, 1.67)			
	None	2.31 (1.97, 2.71)		1.51 (1.28, 1.78)			
Renal ||	Tertiary	1.00 (0.84, 1.19)	285.5	1.00 (0.84, 1.19)	59.2	79	67
	Secondary	1.94 (1.75, 2.15)		1.55 (1.39, 1.72)			
	Primary - Complete	3.11 (2.90, 3.34)		1.92 (1.79, 2.06)			
	Primary - Incomplete	3.59 (3.31, 3.89)		1.78 (1.64, 1.93)			
	None	3.58 (3.19, 4.01)		1.52 (1.35, 1.71)			
Cancer #	Tertiary	1.00 (0.89, 1.12)	7.6	1.00 (0.89, 1.13)	8.3		
	Secondary	1.03 (0.94, 1.13)		1.02 (0.93, 1.12)			
	Primary - Complete	1.10 (1.02, 1.19)		1.07 (0.99, 1.15)			
	Primary - Incomplete	1.13 (1.03, 1.23)		1.07 (0.97, 1.17)			
	None	0.95 (0.83, 1.08)		0.87 (0.76, 1.00)			
Respiratory	Tertiary	1.00 (0.87, 1.15)	29.2	1.00 (0.86, 1.16)	14.3	51	82
	Secondary	1.49 (1.35, 1.65)		1.32 (1.20, 1.46)			
	Primary - Complete	1.48 (1.36, 1.61)		1.19 (1.09, 1.29)			
	Primary - Incomplete	1.47 (1.33, 1.62)		1.09 (0.98, 1.21)			
	None	1.54 (1.35, 1.76)		1.08 (0.94, 1.23)			
Infective	Tertiary	1.00 (0.80, 1.25)	55.1	1.00 (0.79, 1.26)	11.5	79	49
	Secondary	1.56 (1.34, 1.81)		1.39 (1.19, 1.62)			
	Primary - Complete	1.87 (1.66, 2.12)		1.45 (1.28, 1.63)			
	Primary - Incomplete	2.15 (1.87, 2.46)		1.46 (1.27, 1.68)			
	None	2.67 (2.24, 3.17)		1.65 (1.38, 1.97)			
Other/Ill-defined/External	Tertiary	1.00 (0.80, 1.25)	27.5	1.00 (0.80, 1.26)	4.3	84	59
	Secondary	1.14 (0.96, 1.35)		1.05 (0.89, 1.25)			
	Primary - Complete	1.28 (1.11, 1.47)		1.06 (0.92, 1.22)			
	Primary - Incomplete	1.51 (1.29, 1.77)		1.11 (0.95, 1.30)			
	None	2.02 (1.67, 2.43)		1.33 (1.09, 1.62)			

*Adjusted for sex.
† Adjusted for smoking status, alcohol consumption frequency, leisure time physical activity, diabetes status, adiposity (weight, height, waist and hip circumference) and systolic blood pressure.
†† Degrees of freedom: 5.
$ Degrees of freedom: 24.
|| Results are not shown for deaths attributed to acute diabetic crises, because all such deaths were due to diabetes.
Percentage reduction in x^2 and log RR not estimated, because there was little association between education and cancer death.

Table S6. Baseline characteristics of 46 674 men aged 35-74 years by reported income at recruitment

	Income (pesos/month)				
	**None reported (5 031)**	**<1500 (9 062)**	**1500-<3000 (12 628)**	**3000-<4500 (9 866)**	**≥4500 (10 087)**
Age, years †	56.5 (11.3)	56.1 (11.7)	50.7 (10.8)	48.6 (9.7)	47.9 (9.1)
Resident of Coyoacán	1470 (29%)	4321 (48%)	5759 (46%)	3797 (38%)	5236 (52%)
Tertiary education attained	808 (16%)	747 (8%)	1526 (12%)	2528 (26%)	6110 (61%)
Income, pesos/month	0 (0)	917 (397)	2056 (394)	3480 (455)	9350 (9326)
Social Development Index ††	0.74 (0.10)	0.74 (0.09)	0.74 (0.09)	0.75 (0.10)	0.81 (0.11)
Smoking status					
Current smoker	2247 (45%)	4360 (48%)	6748 (53%)	5392 (55%)	5047 (50%)
Ex-smoker	1650 (33%)	3003 (33%)	3537 (28%)	2577 (26%)	2710 (27%)
Never smoker	1134 (23%)	1699 (19%)	2343 (19%)	1897 (19%)	2330 (23%)
Drinking behaviour					
Current drinker	3307 (66%)	6509 (72%)	9811 (78%)	7982 (81%)	8608 (85%)
Former drinker	1238 (25%)	1973 (22%)	2134 (17%)	1387 (14%)	975 (10%)
Never drinker	486 (10%)	580 (6%)	683 (5%)	497 (5%)	504 (5%)
Leisure-time physical activity					
None	3754 (75%)	6846 (76%)	9153 (72%)	6747 (68%)	6033 (60%)
Up to 2 times/week	428 (9%)	855 (9%)	1743 (14%)	1591 (16%)	1763 (17%)
Regular, ≥3 times/week	849 (17%)	1361 (15%)	1732 (14%)	1528 (15%)	2291 (23%)
Anthopometry, blood pressure and HbA1c					
Body mass index, kg/m²	27.7 (4.5)	27.8 (4.3)	28.1 (4.2)	28.2 (4.1)	28.1 (4.0)
Waist circumference, cm	97 (11)	96 (11)	96 (10)	96 (10)	97 (10)
Hip circumference, cm	101 (9)	100 (8)	101 (8)	102 (8)	102 (8)
Systolic blood pressure, mmHg	130 (17)	131 (16)	128 (16)	127 (14)	126 (14)
Diastolic blood pressure, mmHg	85 (10)	85 (10)	85 (10)	84 (10)	84 (9)
HbA1c, % ||	5.7 (1.1)	5.7 (1.1)	5.7 (1.0)	5.6 (1.0)	5.6 (0.9)
Prior diseases #					
Diabetes	1044 (21%)	1461 (16%)	1625 (13%)	1065 (11%)	894 (9%)
Cardiovascular disease	206 (4%)	362 (4%)	296 (2%)	181 (2%)	217 (2%)
Cancer	40 (1%)	67 (1%)	50 (<0.5%)	38 (<0.5%)	50 (<0.5%)
Other **	351 (7%)	537 (6%)	566 (4%)	419 (4%)	455 (5%)

Results shown are frequencies (column percentages) or mean (standard deviation); HbA1c = haemoglobin A1c.
* Same exclusions applied as described in Table 1
† Median (interquartile range) for men: 50.5 years (41.5, 59.5)
†† Higher scores represent higher area-based social development.
$ HbA1c among participants without previously-diagnosed diabetes.
|| Self-reported diseases.
Other diseases include emphysema, chronic kidney disease, peptic ulcer, cirrhosis, and peripheral arterial disease.

Figure S1. Relevance of education to mortality from any cause at ages 35−74 years, by sex

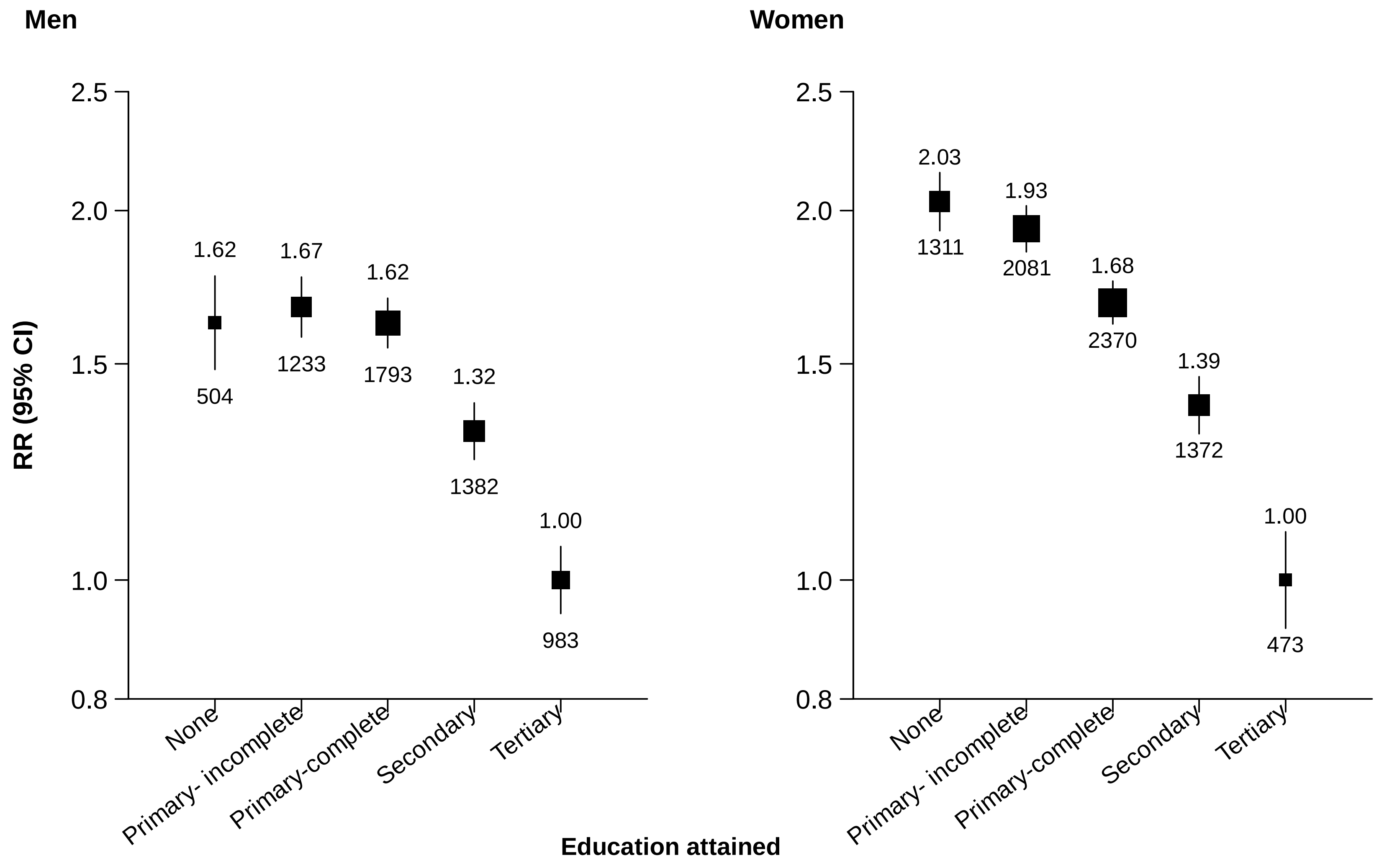

Rate ratios (RRs) are stratified by age-at-risk. The numbers above the squares are the RRs and the numbers below the squares are the number of deaths in that group. The size of each square is proportional to the amount of data available. The error bars represent 95% confidence intervals.

Figure S2. Relevance of education to cause−specific mortality among women at ages 35−74 years

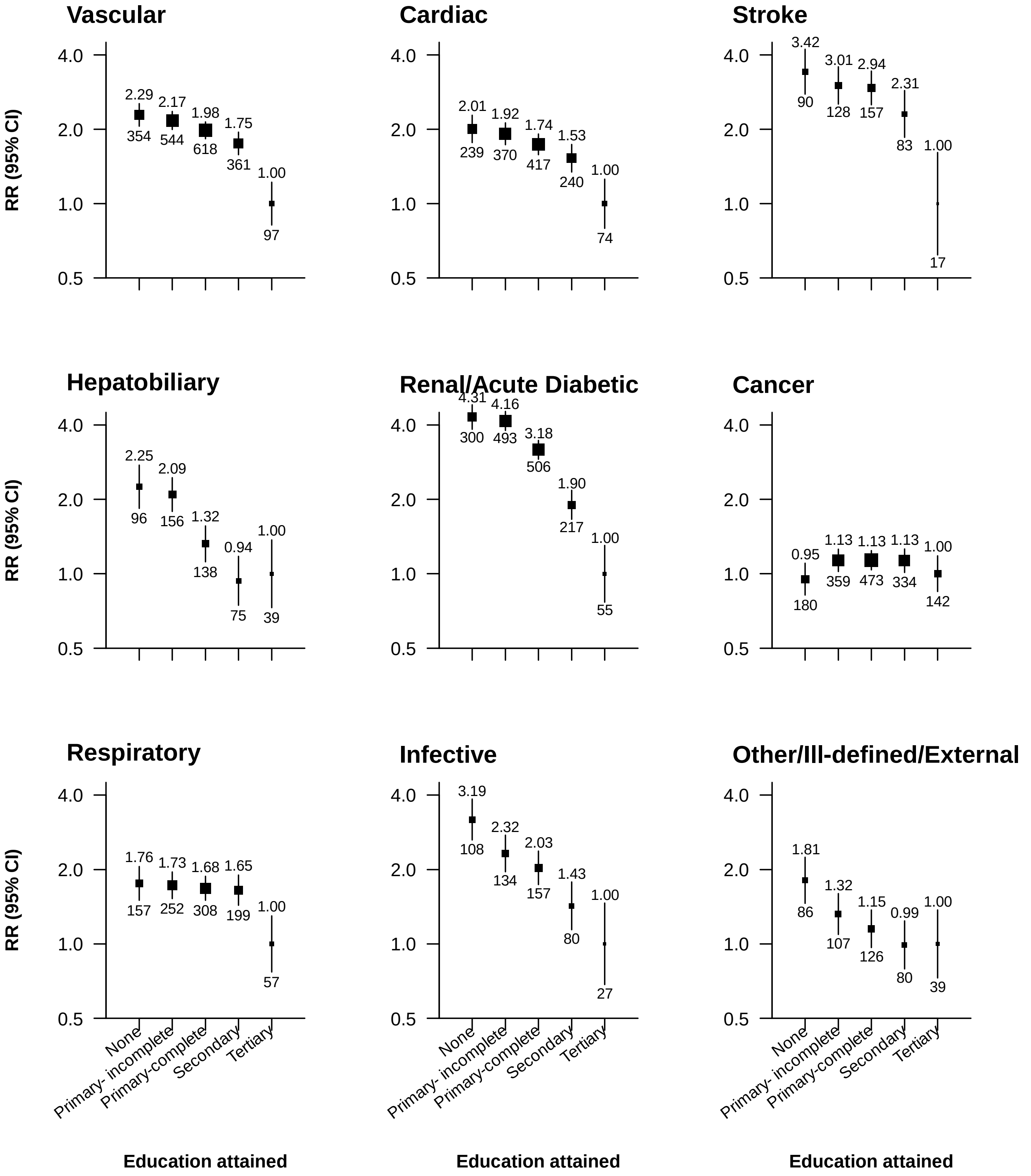

Rate ratios (RRs) are stratified by age-at-risk. The numbers above the squares are the RRs and the numbers below the squares are the number of deaths in that group. The size of each square is proportional to the amount of data available. The error bars represent 95% confidence intervals.

Figure S3. Relevance of education to cause−specific mortality among men at ages 35−74 years

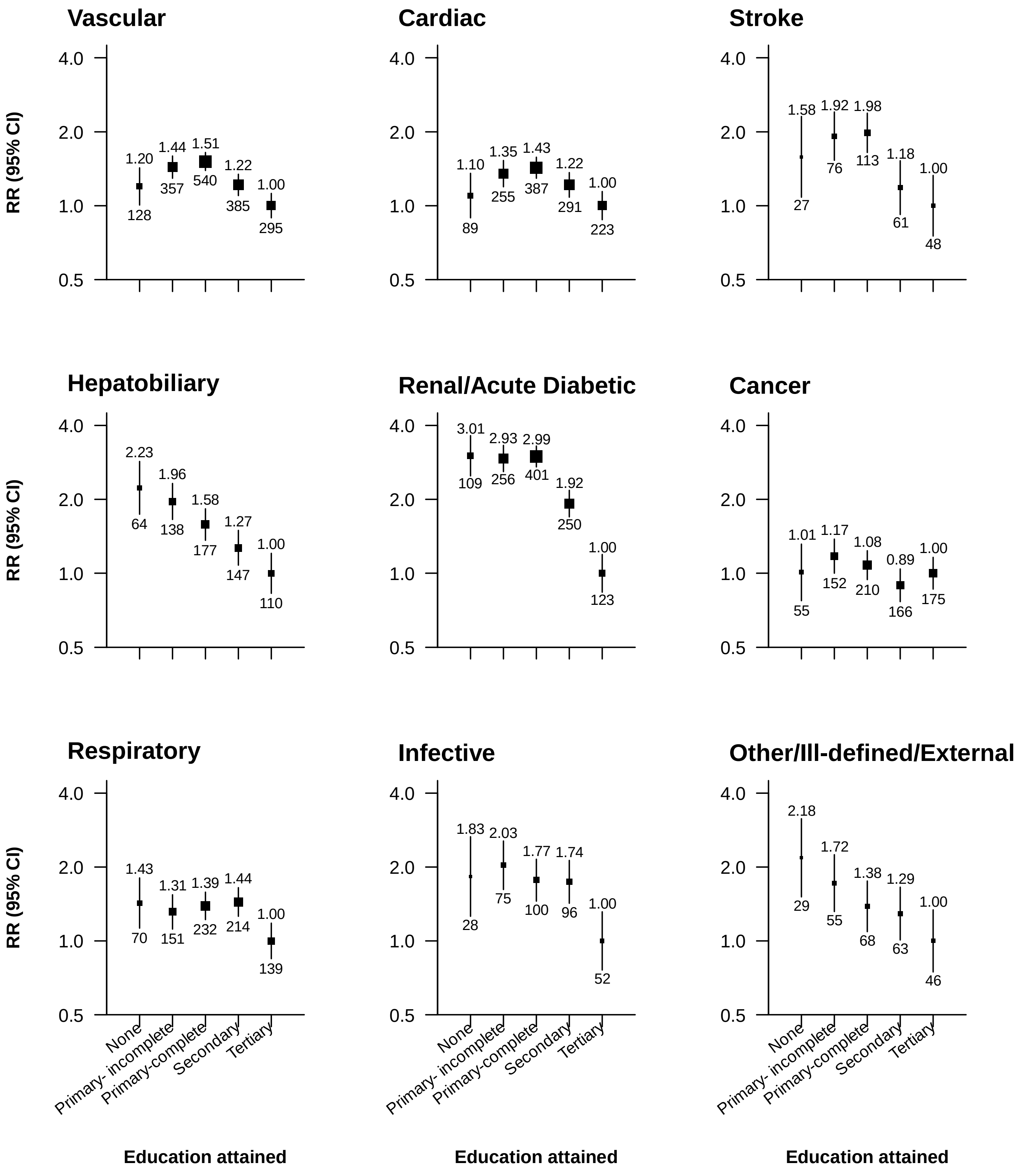

Rate ratios (RRs) are stratified by age-at-risk. The numbers above the squares are the RRs and the numbers below the squares are the number of deaths in that group. The size of each square is proportional to the amount of data available. The error bars represent 95% confidence intervals.

Figure S4. Relevance of education to mortality from any cause at ages 75−84 years

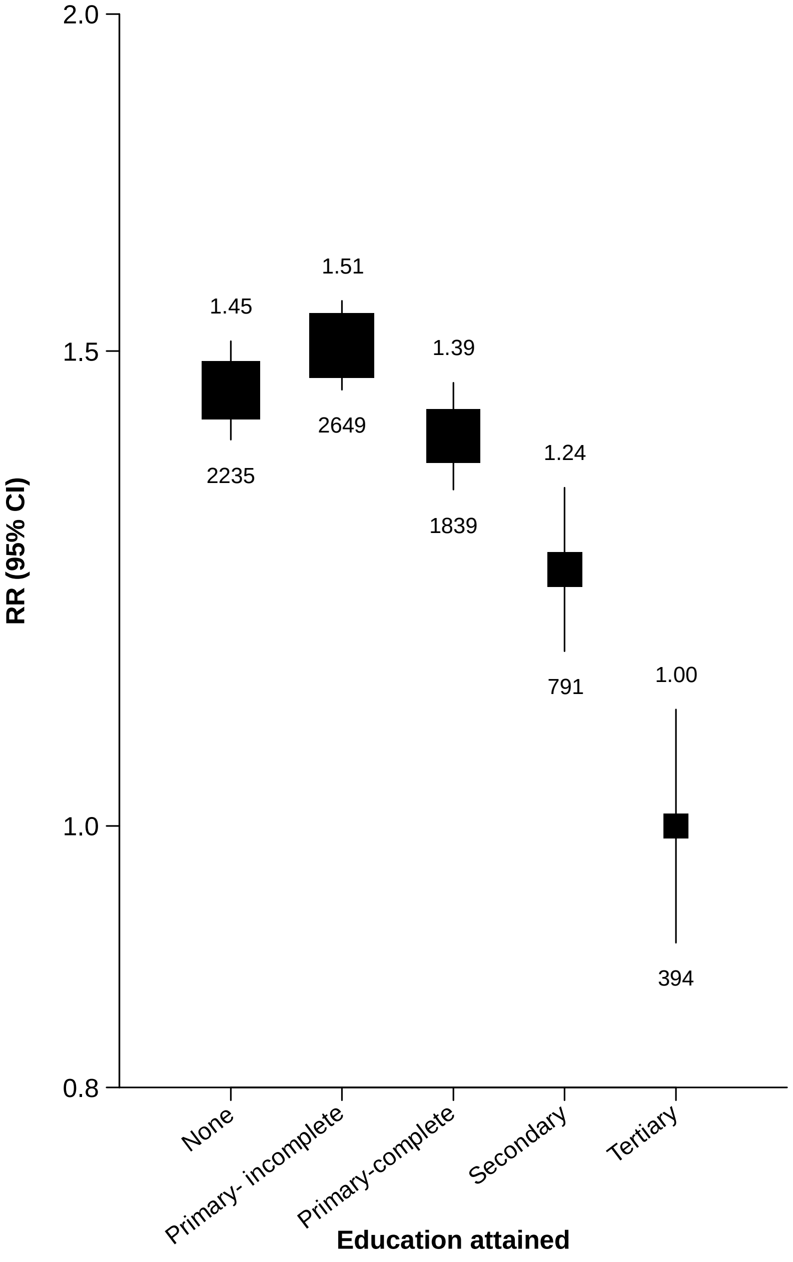

Rate ratios (RRs) are stratified by age-at-risk and sex. The numbers above the squares are the RRs and the numbers below the squares are the number of deaths in that group. The size of each square is proportional to the amount of data available. The error bars represent 95% confidence intervals.

Figure S5. Relevance of education to cause−specific mortality at ages 75−84 years

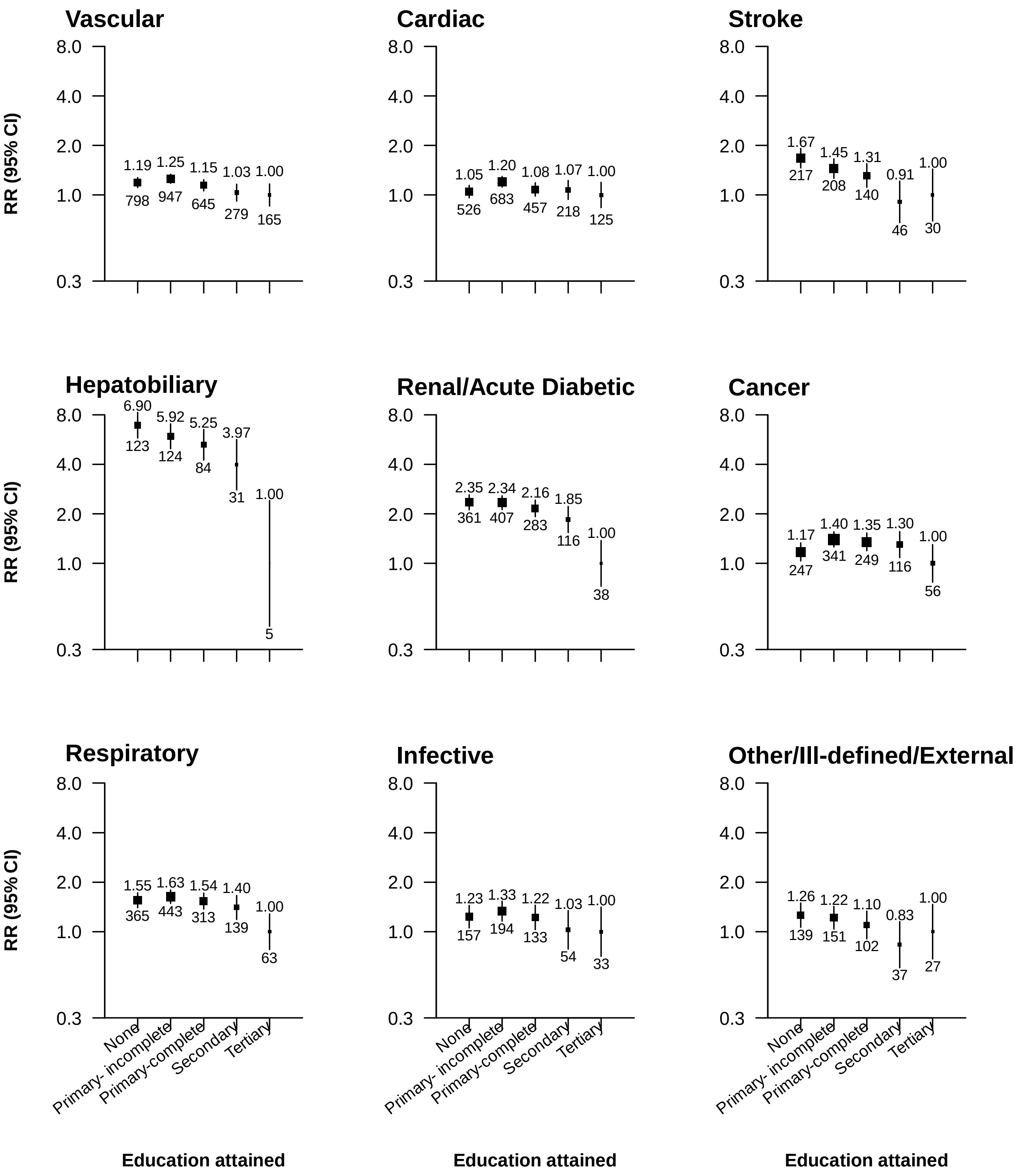

Rate ratios (RRs) are stratified by age-at-risk and sex. The numbers above the squares are the RRs and the numbers below the squares are the number of deaths in that group. The size of each square is proportional to the amount of data available. The error bars represent 95% confidence intervals.

Figure S6. Relevance of income to mortality from any cause among men at ages 35-74 years

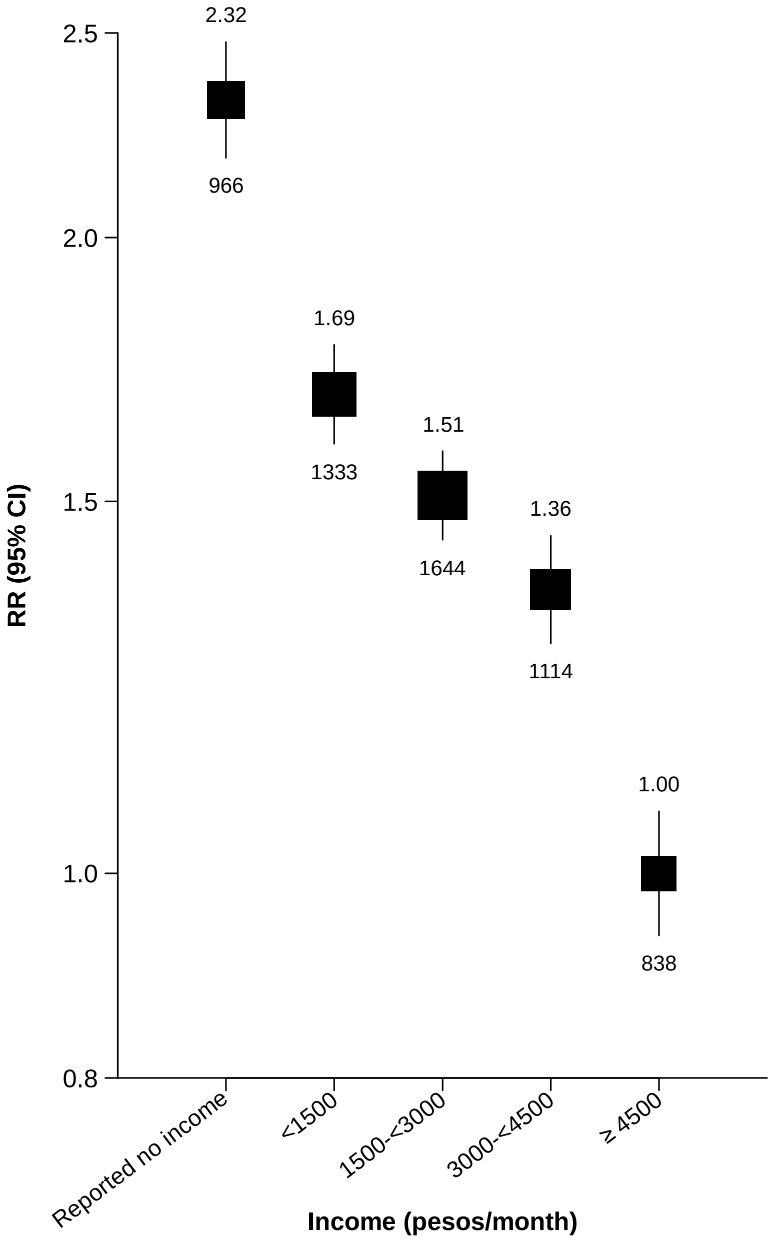

Rate ratios (RRs) are stratified by age-at-risk. The numbers above the squares are the RRs and the numbers below the squares are the number of deaths in that group. The size of each square is proportional to the amount of data available. The error bars represent 95% confidence intervals.

Figure S7. Relevance of income to cause−specific mortality among men at ages 35−74 years

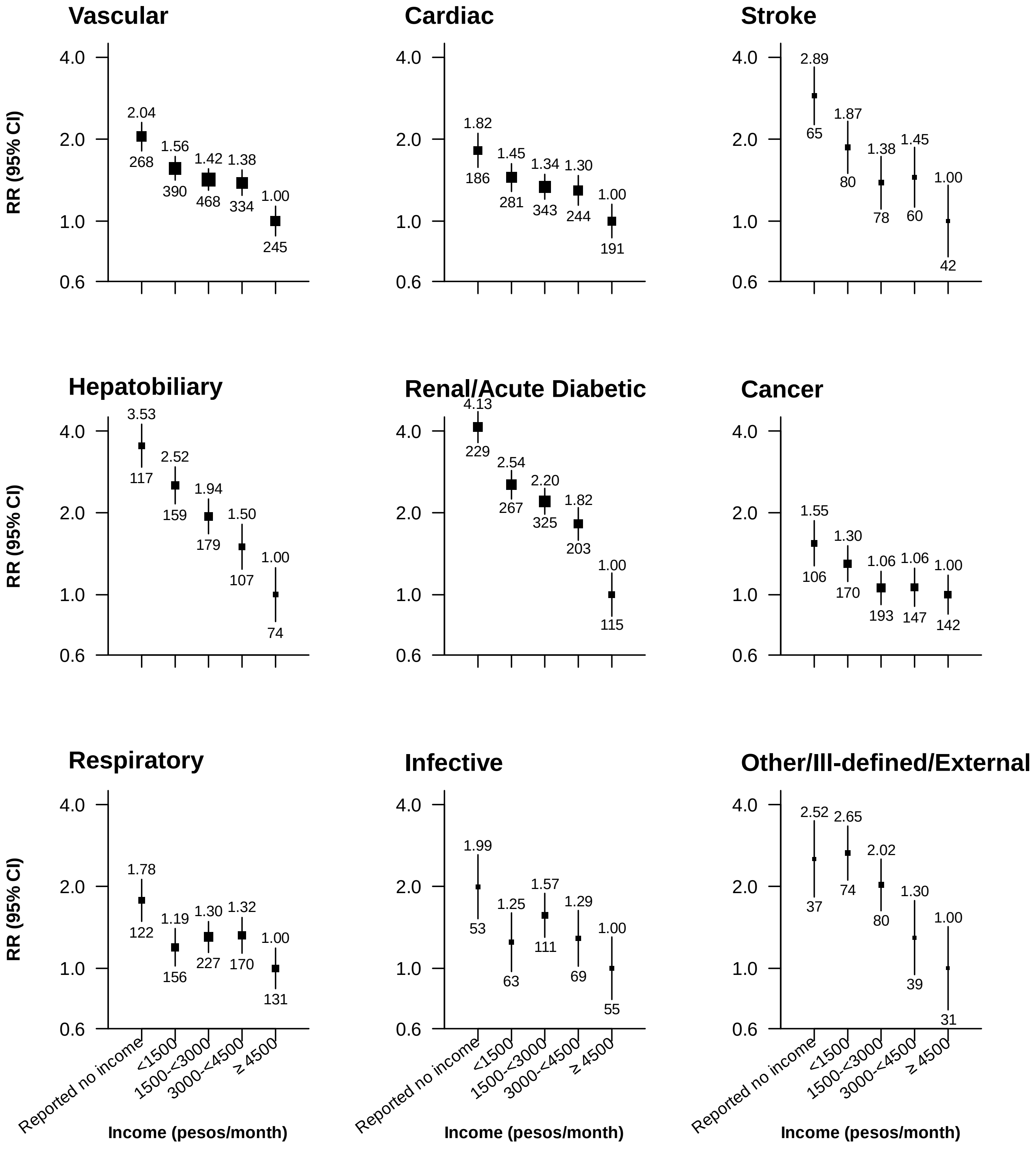

Rate ratios (RRs) are stratified by age-at-risk. The numbers above the squares are the RRs and the numbers below the squares are the number of deaths in that group. The size of each square is proportional to the amount of data available. The error bars represent 95% confidence intervals.

Figure S8. Relevance of Social Development Index to mortality from any cause at ages 35−74 years

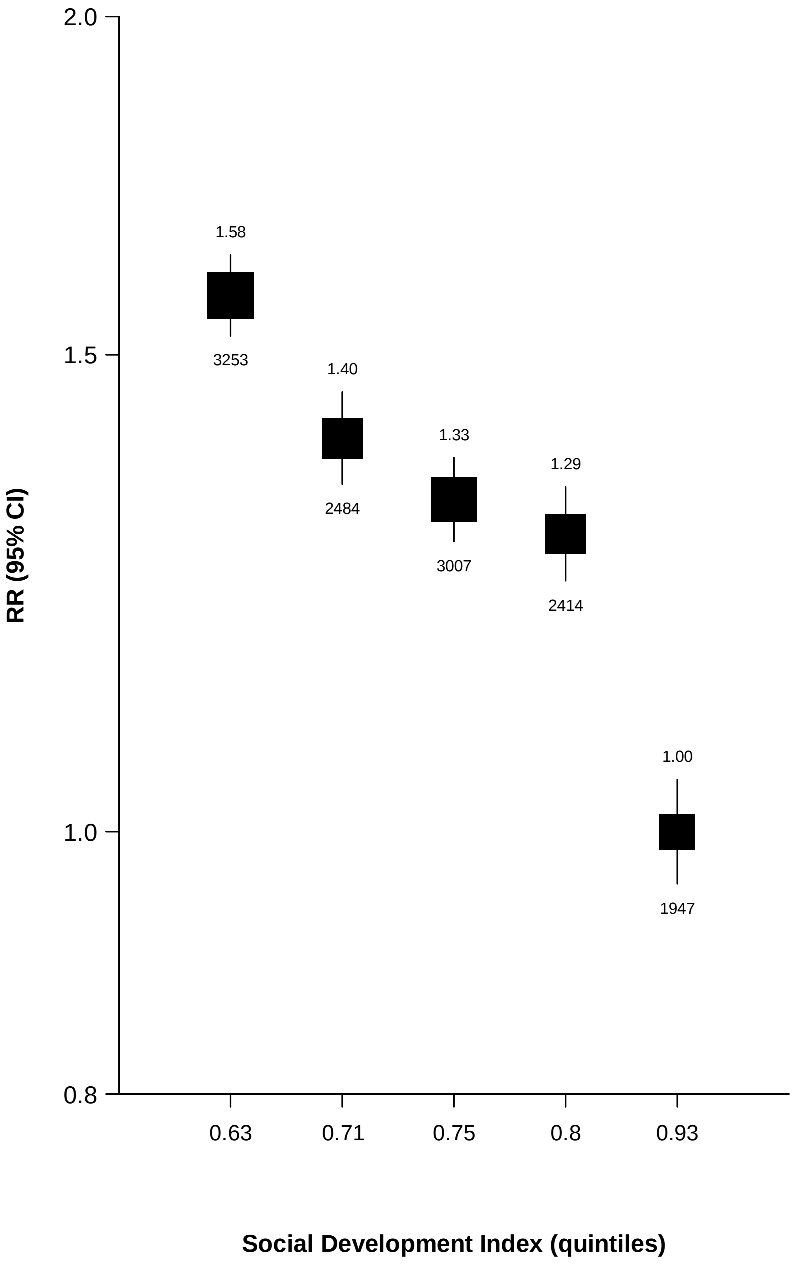

Rate ratios (RRs) are stratified by age-at-risk and sex. The numbers above the squares are the RRs and the numbers below the squares are the number of deaths in that group. The size of each square is proportional to the amount of data available. The error bars represent 95% confidence intervals.

Figure S9. Relevance of Social Development Index to mortality from any cause at ages 35−74 years, by sex

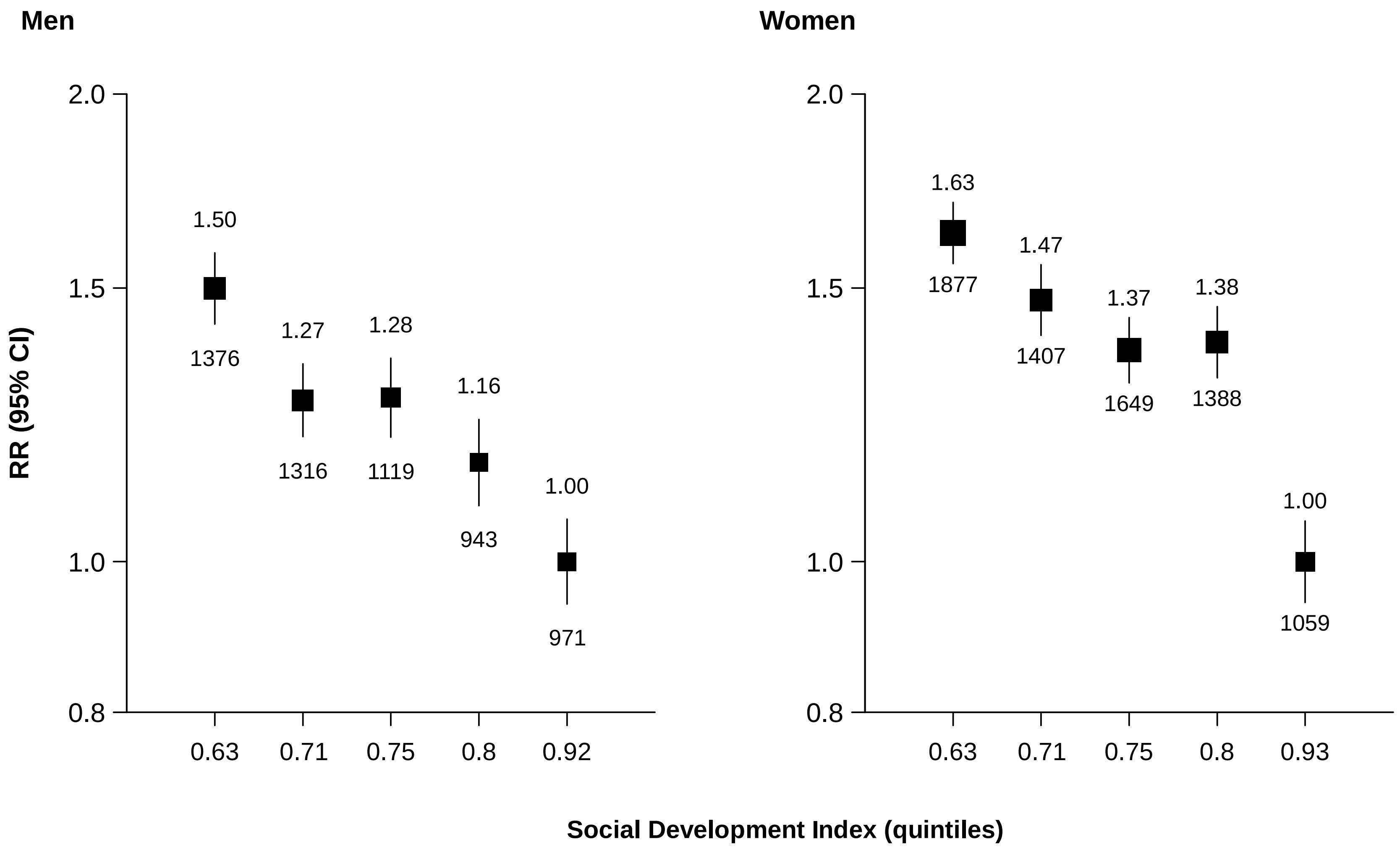

Rate ratios (RRs) are stratified by age-at-risk. The numbers above the squares are the RRs and the numbers below the squares are the number of deaths in that group. The size of each square is proportional to the amount of data available. The error bars represent 95% confidence intervals.

Figure S10. Relevance of Social Development Index to cause−specific mortality at ages 35−74 years

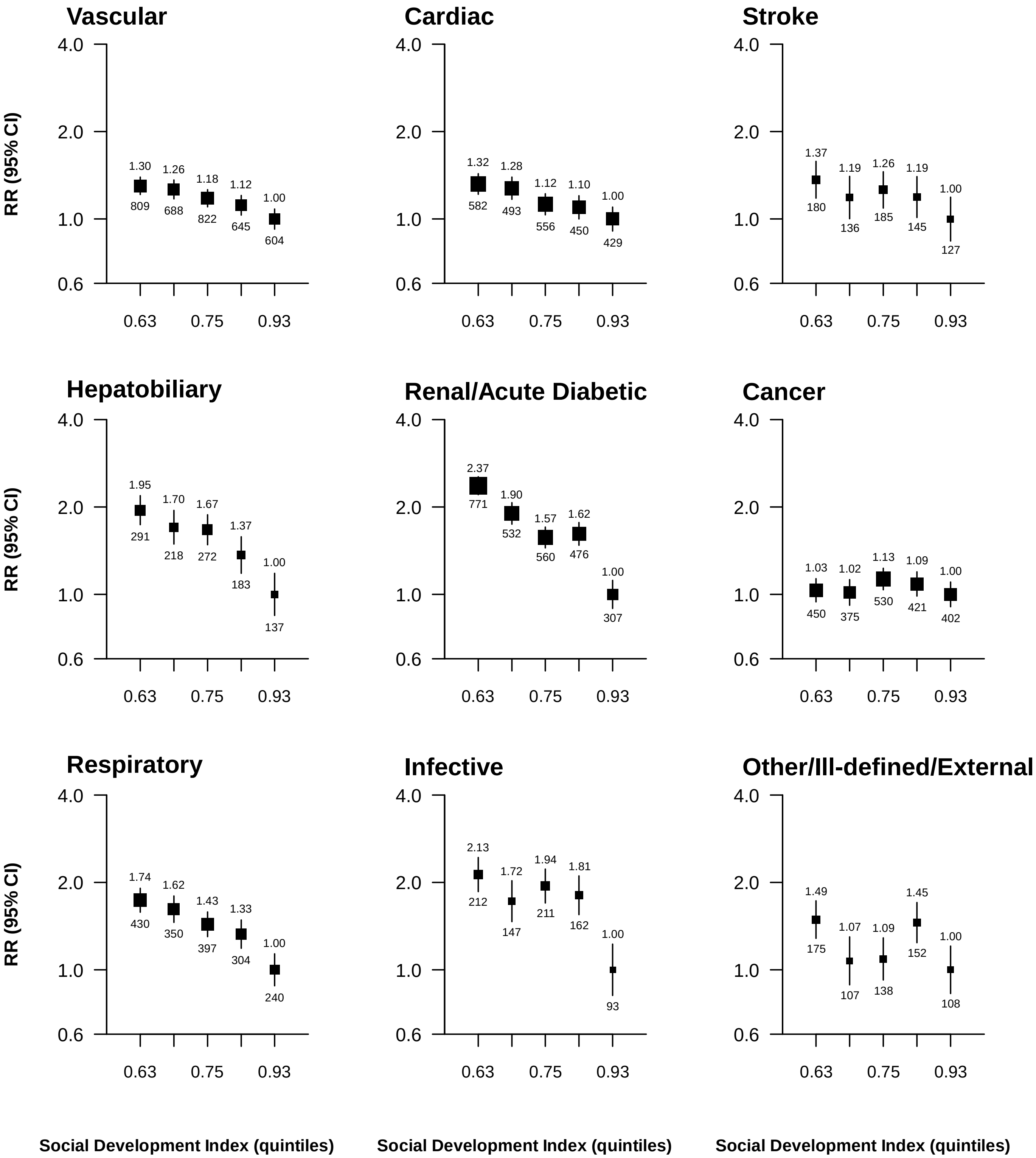

Rate ratios (RRs) are stratified by age-at-risk and sex. The numbers above the squares are the RRs and the numbers below the squares are the number of deaths in that group. The size of each square is proportional to the amount of data available. The error bars represent 95% confidence intervals.
